# A detailed methodology for the long-term *in vitro* culture and analysis of three-dimensional, self-structuring bone models generated from cell lines or primary osteoblastic cell populations

**DOI:** 10.12688/f1000research.130779.2

**Published:** 2024-05-07

**Authors:** Melissa Finlay, Laurence A Hill, Georgiana Neag, Binal Patel, Miruna Chipara, Hannah C Lamont, Kathryn Frost, Kieran Patrick, Jonathan W Lewis, Thomas Nicholson, James Edwards, Simon W Jones, Liam M Grover, Amy J Naylor

**Affiliations:** 1 Healthcare Technologies Institute, University of Birmingham, Birmingham, West Midlands, B15 2TT, UK; 2 Institute of Inflammation and Ageing, University of Birmingham, Birmingham, West Midlands, B15 2TT, UK; 3 NDORMS, University of Oxford, Oxford, Oxfordshire, OX3 7HE, UK

**Keywords:** Osteocyte, bone, animal reduction, 3D model, in vitro culture

## Abstract

**Background:**

There are insufficient *in vitro* bone models that accommodate long-term culture of osteoblasts and support their differentiation to osteocytes. The increased demand for effective therapies for bone diseases, and the ethical requirement to replace animals in research, warrants the development of such models.

Here we present an in-depth protocol to prepare, create and maintain three-dimensional, *in vitro*, self-structuring bone models that support osteocytogenesis and long-term osteoblast survival (>1 year).

**Methods:**

Osteoblastic cells are seeded on a fibrin hydrogel, cast between two beta-tricalcium phosphate anchors. Analytical methods optimised for these self-structuring bone model (SSBM) constructs, including RT-qPCR, immunofluorescence staining and XRF, are described in detail.

**Results:**

Over time, the cells restructure and replace the initial matrix with a collagen-rich, mineralising one; and demonstrate differentiation towards osteocytes within 12 weeks of culture.

**Conclusions:**

Whilst optimised using a secondary human cell line (hFOB 1.19), this protocol readily accommodates osteoblasts from other species (rat and mouse) and origins (primary and secondary). This simple, straightforward method creates reproducible *in vitro* bone models that are responsive to exogenous stimuli, offering a versatile platform for conducting preclinical translatable research studies.

##  

Research highlights **Scientific benefits**

Allows:

• Study of osteoblast activity in long-term culture (>8 weeks).
• Study of osteoblast to osteocyte differentiation.
• Study of mineralisation and extracellular matrix production by osteoblasts.
• Study of osteoblasts and osteocytes in a 3D, organotypic environment.

**3Rs benefits**

• Reduces the need for osteoblast and osteocyte isolation from vertebrates in skeletal research by supporting long-term culture of primary osteoblasts and secondary cell lines.
• Provides a platform for osteocyte research *in vitro*, reducing the requirement for vertebrate *in vivo* experimentation.

**Practical benefits**

• Inexpensive, uses commonly available equipment and reagents.
• Readily adaptable - can use cells of different origin and species.
• Organotypic - 3D environment and matrix production and mineralisation performed by the osteoblasts.

**Current applications**

• Suitable for comparing phenotypes of osteoblasts isolated from genetically modified vertebrates or from patient samples.
• Suitable for studying osteoblast and osteocyte function and for screening their response to therapeutic candidate compounds.
• Suitable for long-term 3D culture of human osteoblast cell lines, such as hFOB, and for assessment and manipulation of their differentiation towards osteocytes.

**Potential applications**

Offers a platform for:

• Studying osteoblast/osteocyte interactions with other cell types e.g. osteoclasts.
• Screening of compound libraries to identify therapeutic candidate compounds.
• Assessing efficacy of drugs and therapeutics under relevant conditions.

## Introduction

Bone is a dynamic tissue that continually adapts throughout life to ensure maintenance of strength and integrity and to fulfil its many other functions, which include: adaptation to loading, preservation of mineral homeostasis (particularly calcium and phosphorous) and conservation of energy balance. ^1^ These roles are made possible by the tightly controlled intercellular process called bone remodelling. Bone remodelling is a cyclical process that begins with tissue breakdown by bone resorbing osteoclasts, to release stored mineral and osteogenic factors such as bone morphogenic proteins and fibroblast growth factors. ^2^ Resorption is followed by the formation of new bone tissue by osteoblasts. The balance between bone formation and bone resorption activity is critical for maintenance of tissue integrity. Likewise, this remodelling process allows bone to be repaired when damaged and to adapt rapidly to changes in physical demand, for example increased weight-bearing sports training or, oppositely, unloading through bed rest after injury and illness ^3^ as well as from prolonged zero gravity experienced during space exploration. ^4^

The precise coupling of osteoblast and osteoclast activity is tightly regulated by osteocytes (reviewed in Ref. 5 ). Up to 20% of osteoblasts differentiate into osteocytes ^6^^,^^7^ which become entombed within newly deposited, immature bone tissue. Upon differentiation, they undergo a profound morphological change from a polygonal structure to a highly dendritic form. Entombed within individual lacunae, osteocyte dendrites extend through canaliculi to connect with other osteocytes, osteoblasts, osteoclasts, neurons, and the vasculature, creating a highly interconnected osteocyte-canalicular network. This distinguishing feature of osteocytes is a stipulation for their role as the main regulatory unit in bone metabolism and as orchestrators of bone cell activity. ^7^^,^^8^

Dysregulation of bone remodelling is caused by multiple factors, the most notable of which is aging, which results in osteoporosis. As the global population ages, osteoporosis-induced fragility fractures are predicted to increase markedly – estimates made from six of the largest countries in Europe put the rise at 23.3% by 2030 compared to 2017. ^9^ These figures give heightened urgency and a major push in research efforts to meet, or better yet curb, the impending demands for effective bone therapeutics.

Currently, bone researchers employ a range of platforms within *in vivo*, *ex vivo* and *in vitro* systems, all with their own advantages and disadvantages - detailed reviews of these platforms have been reported recently. ^10^^,^^11^ *In vivo* bone models provide a complete and vascularised osseous system used to investigate bone metabolism, disease, healing, and development, as well as drug candidate efficacy and safety. However, these studies often require large sample numbers for moderate to severe procedures such as bone healing and ovariectomy-induced osteoporosis. Although *ex vivo* bone models, such as calvarial and femoral head culture, can be employed to investigate some of the same research questions as *in vivo* studies, the high running costs and intrinsic interspecies differences that exist when using animal tissue to study human disease limit their utility. Osteoblast and osteoclast *in vitro* cultures are widely employed, with multiple activity assessment assays and complex co-culture systems developed. However, they have been limited by the minimal success of including osteocytes within these systems, and by the challenges of recapitulating the composition of inorganic mineral amalgamated to a complex hierarchy of collagen fibres that characterises native bone tissue in an *in vitro* system. ^12^

To obtain a complete reflection of bone metabolic activity, the presence of interconnected osteocytes within any bone model is fundamental, however, isolation of primary osteocytes followed by cell culture under standard conditions is not successful and results in dedifferentiation to osteoblasts. ^13^ Some immortalised osteocytic cell lines have been developed, which retain osteocyte morphology during 2D culture, for example the murine osteocyte cell line, ^14^ MLO-Y4 ^14^ and the human preosteocytic cell line, HOB-01-C1 ^15^. Although, these cell lines share common characteristics specific to *in vivo* osteocytes (such as low ALP activity and high osteocalcin production, MLO-Y4 has uncharacteristically low sclerostin production and the requirement of interferon-gamma to maintain the immortalised status of HOB-01-C1 limits its use in co-culture with osteoblasts and osteoclasts. The challenges of *in vitro* osteocyte culture have led researchers to conduct a significant amount of bone research *in vivo*, with murine models being preferred due to lower running costs, simpler husbandry and genetic manipulability, ^16^^,^^17^ as well as their rapid rate of bone turnover. ^18^ In recent years, the movement towards replacing and reducing reliance on laboratory animals, and the requirement for *in vitro* systems for compound screening in drug development pipelines that are translatable, have renewed interest in the development of humanised *in vitro* bone assays capable of supporting osteoblasts and their differentiation towards osteocytes.

**Figure 1.**
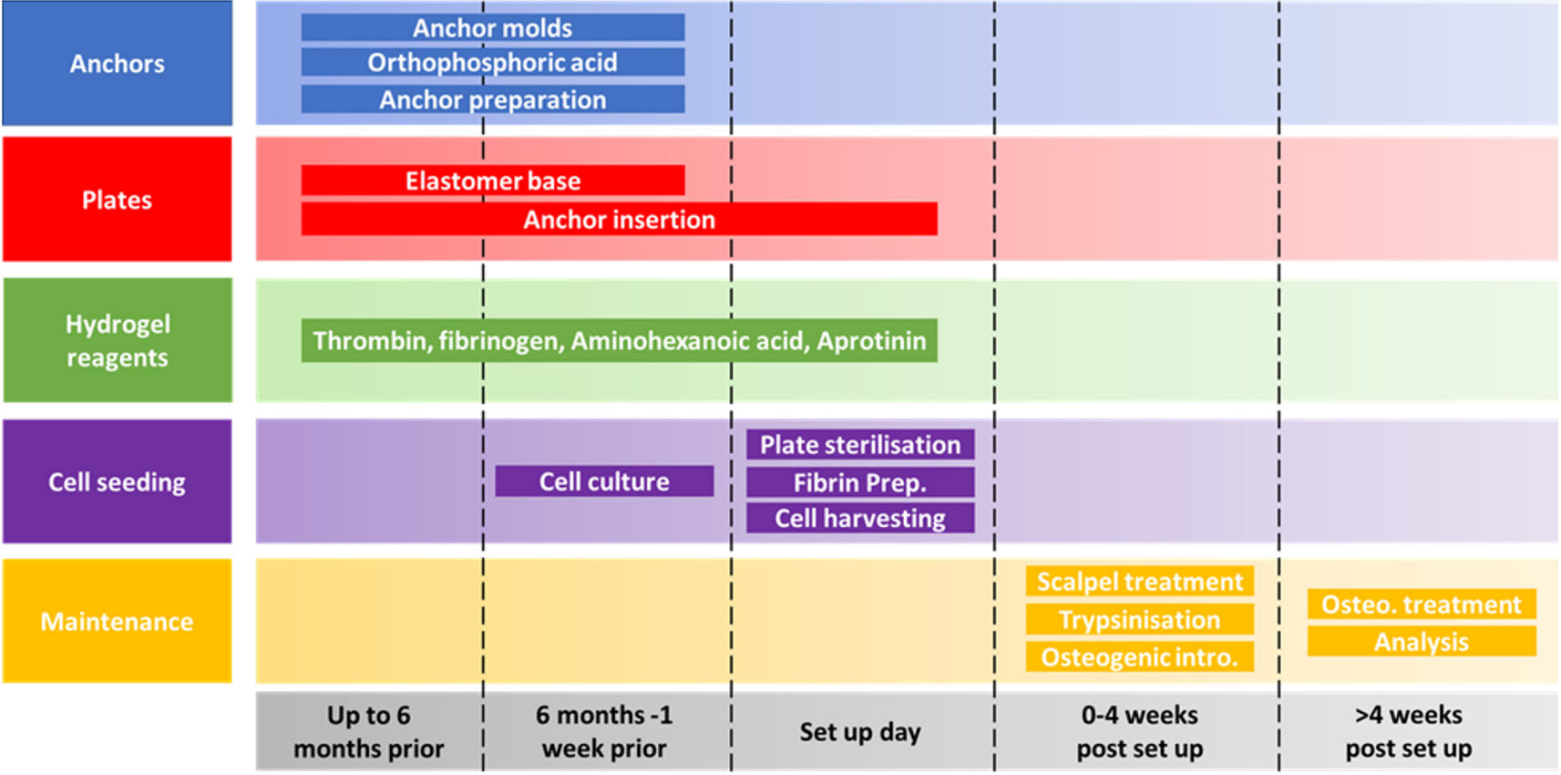
Schematic of the self-structuring bone model (SSBM) method planning and maintenance time scales. The SSBM method is divided into 5 separate aspects (anchors, plates, hydrogel reagents, cell seeding and maintenance) each with their own steps for preparing, setting up and maintaining SSBM constructs. The minimum amount of preparation time required for setting up SSBM cultures is one week, providing all materials are available. However, many of the steps can be carried out up to six months in advance; this is recommended for stock preparation. Depending on the cell lines/types to be used for SSBM set up, preparing cells for cell seeding can vary and may take less than one week (i.e. faster proliferation rates). Cell seeding procedures on set up day will take 1-2 hours to complete. Maintenance steps within the first four weeks post SSBM set-up are performed on defined days after cell seeding but may vary depending on the cell type chosen. Analytical timepoints are selected at the researchers’ discretion, and dependent on the research question, with four weeks post SSBM set-up (i.e. just prior to introducing and maintaining osteogenic treatment) typically being the minimum analytical timepoint.

Recent advances in 3D cell culture systems and tissue engineering have seen an exponential increase in the creation and use of *in vitro* platforms that recapitulate *in vivo* tissue microenvironments. Cells cultured under these conditions have *in vivo*-like responses due to having a more natural conformation unlike 2D platforms. As such, efforts have been made to create bone models that capture the native tissue both physiochemically and biologically. Here we describe a three-dimensional, self-structuring bone model (SSBM) culture system that uses a biocompatible scaffold to support the differentiation of osteoblasts and the production of a complex collagen-rich mineralised matrix, that resembles native bone. Unlike other bone models that are available (reviewed in Refs. 10, 11), our method does not require specialised equipment or bespoke bioreactors and can be maintained under standard cell culture conditions (specific to the cell line of choice). Its unique in-built strain and source of calcium phosphate provided by two anchorage points simulates the *in vivo* tissue microenvironment to support collagen-containing matrix production by osteoblasts, as well as their long-term survival and differentiation (> 1 year) towards osteocytogenesis, along with mineralisation of the newly produced matrix – a combination of properties currently lacking with the other *in vitro* bone models available. We provide a detailed and descriptive protocol for preparing, setting up and maintaining SSBM constructs [ Figure 1] and the analytical methods that have been developed specifically for their analysis.

## Methods

### Choice of cell source

This method (originally described in Ref. 19) relies on osteoblast-mediated contraction of a biocompatible scaffold (fibrin hydrogel) around a source of calcium and phosphate ions in the form of two cement anchors consisting of a combination of brushite (CaHPO _4_.2H _2_O) and B-TCP (Ca _3_(PO _4_) _2_). Contraction provides a 3D environment within which the osteoblasts, over time, replace the original fibrin matrix with organotypic collagen and facilitate its calcium phosphate mineralisation.

The original method ^19^^,^^20^ utilised primary rat periosteal cells. The figures included here were generated using the human osteoblastic cell line, hFOB 1.19 (RRID: CVCL_3708) – selected based on their homogenous human origin, as well their stable karyotype, ^21^ but the SSBM method can be readily adapted to accommodate osteoblasts derived from a number of different species, from primary osteoblasts isolated from vertebrate bone samples or from cell lines. We have used primary human osteoblasts and the human osteoblast cell line, hFOB 1.19, ^22^ with similar success (data not shown). We note that primary cells have high levels of batch-batch variation in cell type populations and metabolic rates, as has been previously observed ^23^ and that there are intrinsic differences in the proliferation rates of cell lines therefore, seeding densities and culture media composition should be adapted and validated on a case-by-case basis.

The following preparation, set up and maintenance methods have been separated into 5 distinct aspects, summarised in Figure 1.

**1. Anchor preparation**

**Note – Sections 1.1-1.3 are prepared under non-sterile conditions**

*1.1 Anchor mould preparation*

Required:

• Fused deposition modelling 3D Printer
• Fusion 360 computer-aided design software (V.2.0) (Supplier: Autodesk, USA), or similar
• 1.75 mm polylactic acid plastic filament (cat no.: 55318; supplier: Verbatim, Germany)
• Perfection Plus Impress-E putty (Soft) (cat no.: 238-0084; supplier: Dental Sky Wholesaler Ltd., Kent (UK only))

**Figure 2.**
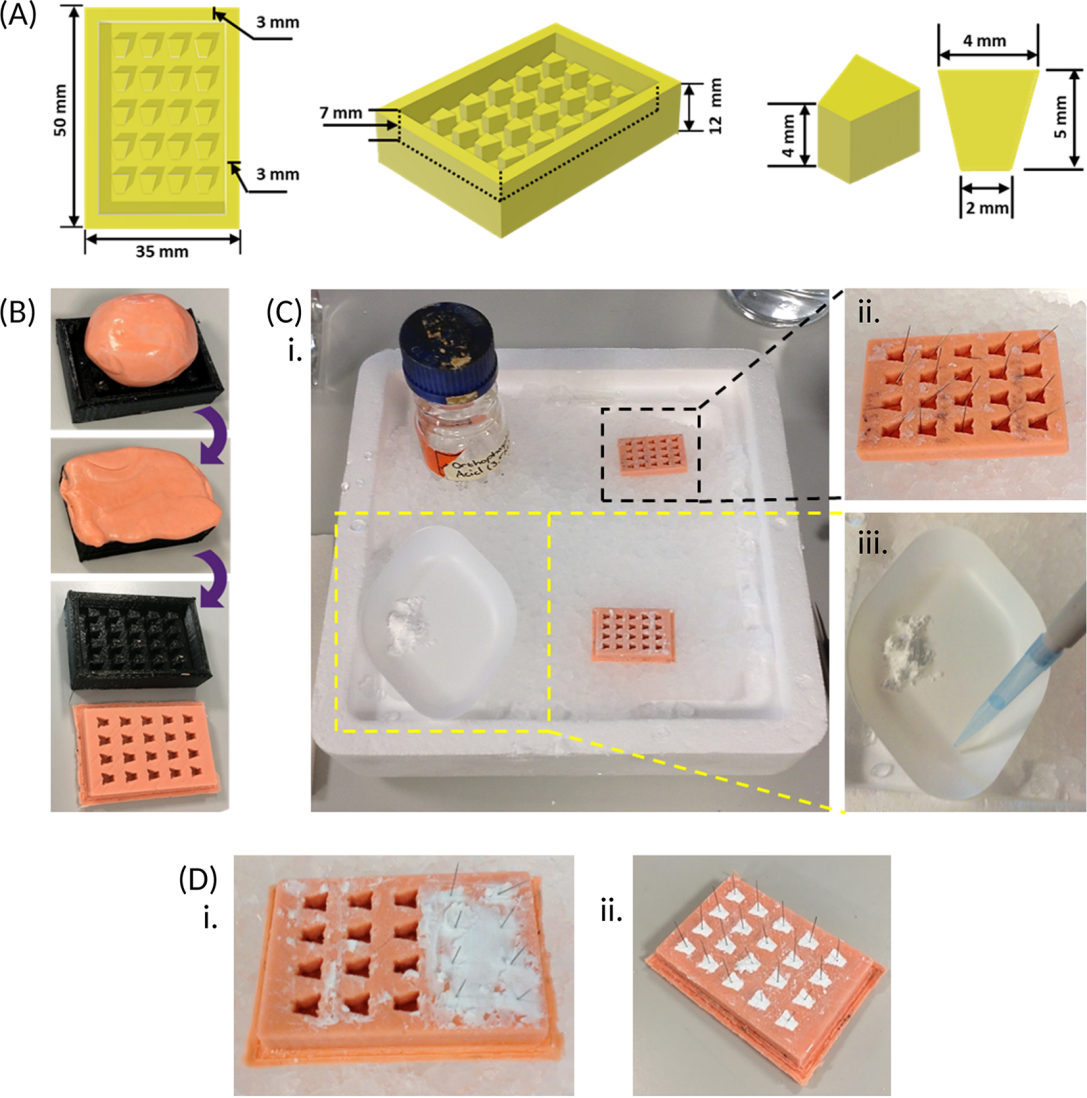
Preparing and using a mould for creating beta-tricalcium phosphate (β-TCP) cement anchors. (A) A 3D sketch of a positive frame for the anchor mould, containing twenty equally spaced pegs, is drawn up with the dimensions provided above, using computer-aided design software, and a reusable frame is 3D printed with polylactic acid. (B) Dental impression material is pressed into the positive frame, ensuring the whole frame is filled, after which the mould is peeled out once completely set. (C) The recommended set up when preparing the β-TCP cement anchors. An ice tray is prepared with a spare anchor mould containing a single insect pin in each well placed in a top corner (black dotted box) and an empty mould in the adjacent bottom corner; as well as the orthophosphoric acid (3.5 M) in the vacant top corner. The β-TCP is weighed out and kept to one side of the weighing boat and placed in the vacant bottom corner of the ice tray (yellow dotted box) at a slight forward angle. The orthophosphoric acid is added to the same weighing boat as the β-TCP but kept separate prior to mixing. (D) When mixed, the resultant cement created, using the weight and volumes outlined [Section 1.3] is sufficient to fill 2-3 rows of the empty anchor mould, and pins from the spare anchor mould (C, black dotted box) are inserted immediately, prior to hardening (i). After repeating cement preparation, the cement is left to completely set within the anchor mould for approximately 3 days (ii).

Steps:

1. 3D print a positive frame of 1.75 mm polylactic acid plastic for the anchor moulds with the dimensions shown [ Figure 2.A]. *3D Object (.3mf) and GCODE files are available as extended data.* ^39^
  ○ *Note - if unable to 3D print on site, there are several professional online printing services who can print the frame quickly and cost effectively from the 3D object file. Recommendation to print 1-3 frames.*
  ○ *Note - once printed, positive frames can be reused multiple times (until anchor shape has become distorted). Shelf life: >4 years.*
2. Prepare the impression putty, as per the supplier’s instructions, and press the putty into the positive frame, ensuring the frame is completely filled.
3. Allow the impress material to set for at least 24 hours at room temperature before removing from the mould. Make at least two. These are the anchor moulds for use in section 1.3.
  ○ *Note - anchor moulds can be reused several times until ripped or shape becomes distorted. Ensure to remove as much excess cement as possible from the mould between uses.*

*1.2 Orthophosphoric acid (3.5 M) preparation*

Required:

• Orthophosphoric acid (85-90% purity) (cat no.: 20624.262; supplier: VWR Chemicals, Leicestershire, UK)
• Distilled water (d.H _2_O)

Steps:

1. Assuming 87.5% purity; for every 100 mL of Orthophosphoric acid:
$$76.8\;\mathrm{mL}\;d.H_{2}O+23.2\;\mathrm{mL}\;\text{Orthophosphoric acid}$$
***CRITICAL - Add the acid to water, never water to acid***
2. Store at 2-4 °C for up to 6 months.

*1.3 Calcium phosphate anchor preparation*

Required:

• Beta-tricalcium phosphate (β-TCP) (<125 μm particle size) (Plasma Biotal Limited., Tideswell, UK)
• Orthophosphoric acid (3.5 M), prepared as described above [Section 1.2]
• Austerlitz insect pins (stainless steel, 0.200 mm ø.) (cat no.: 26002-20; supplier: InterFocus Ltd., Linton, UK)
• Anchor moulds (at least 2) [Section 1.1]
• Fine metal tweezers
• Metal Chattaway or flat-headed spatulas (~10 mm width)
• Large weighing boats (100 mL) (cat no.: 611-9189; supplier: VWR)
• Tray/bucket of ice
• Paper towels

**Note - The recommended workspace set-up is shown [** Figure 2.C.i **] to ensure required equipment is within reach during anchor preparation.**

Steps:

1. Using tweezers, add a single pin to each well of one of the spare anchor moulds [ Figure 2.C.ii].
2. Weigh out 1.25 ± 0.005 g of β-TCP into a large weighing boat (keep the powder to one side of the weighing boat [ Figure 2.C.iii] then place the weighing boat on top of the ice tray.
3. Add 500 μL orthophosphoric acid to the same weighing boat, but away from the powder [ Figure 2.C.iii].
  • *Tip - Point the weighing boat at a small angle down towards you.*
4. Mix the powder and acid together using the metal spatula, keeping the weighing boat on the ice, until the liquid mixture becomes a runny paste.
  • ***Note – there are batch-batch differences with β-TCP powder. Weigh out the mass stated above in [step 1] and adjust the volume of orthophosphoric acid by 100 μL increments/reductions until achieving the desired consistency (e.g. condensed milk).***
5. Quickly scrape and apply the paste onto 2-3 rows of the empty mould using the spatula, then submerge the spatula into the ice, followed by quickly transferring a single pin from the spare mould to each filled well. This must be completed before the cement hardens, which will take about 30 s [ Figure 2.D.i].
  • ***Note – there will be an excess of cement on top of the mould during application, try to scrape off as much as possible, but a slight excess is permissible.***
  • *Tip – plunging the spatula into the ice helps with subsequent cleaning with a paper towel. If the excess cement is not readily removed with wiping, use hot soapy water.*
6. Clean the spatula (as above), then repeat steps 2-5 [Section 1.3] with the remaining wells in the mould, using a new weighing boat each time.
7. Allow the anchors to set in the mould at room temperature for up to 3 days [ Figure 2.D.ii], then remove from the mould and store in a sealed container at room temperature for up to 6 months.

**2. Plate preparation**

***Note – Plates may be prepared under non-sterile conditions, as they are sterilised just prior to hydrogel preparation.***

Required:

• 6-well plate(s) (Cat no.: CLS3516; supplier: Corning, Flintshire, UK) – *Tissue culture-treated plates are recommended, but not essential*
• Sylgard 184 silicone elastomer kit (Cat no.: 634165S; supplier: VWR)
• Calcium phosphate anchors (12 per plate) prepared as described above [Section 1.3]
• Large weighing boats
• BD Emerald disposable syringe (10 mL) (Cat no.: 15205093; supplier: Fisher Scientific, Loughborough, UK)
• BD PlastiPak disposable syringes (1.0 & 50 mL) (Cat nos.: 15489199 & 12651406; supplier: Fisher Scientific)
• Tweezers

**Figure 3.**
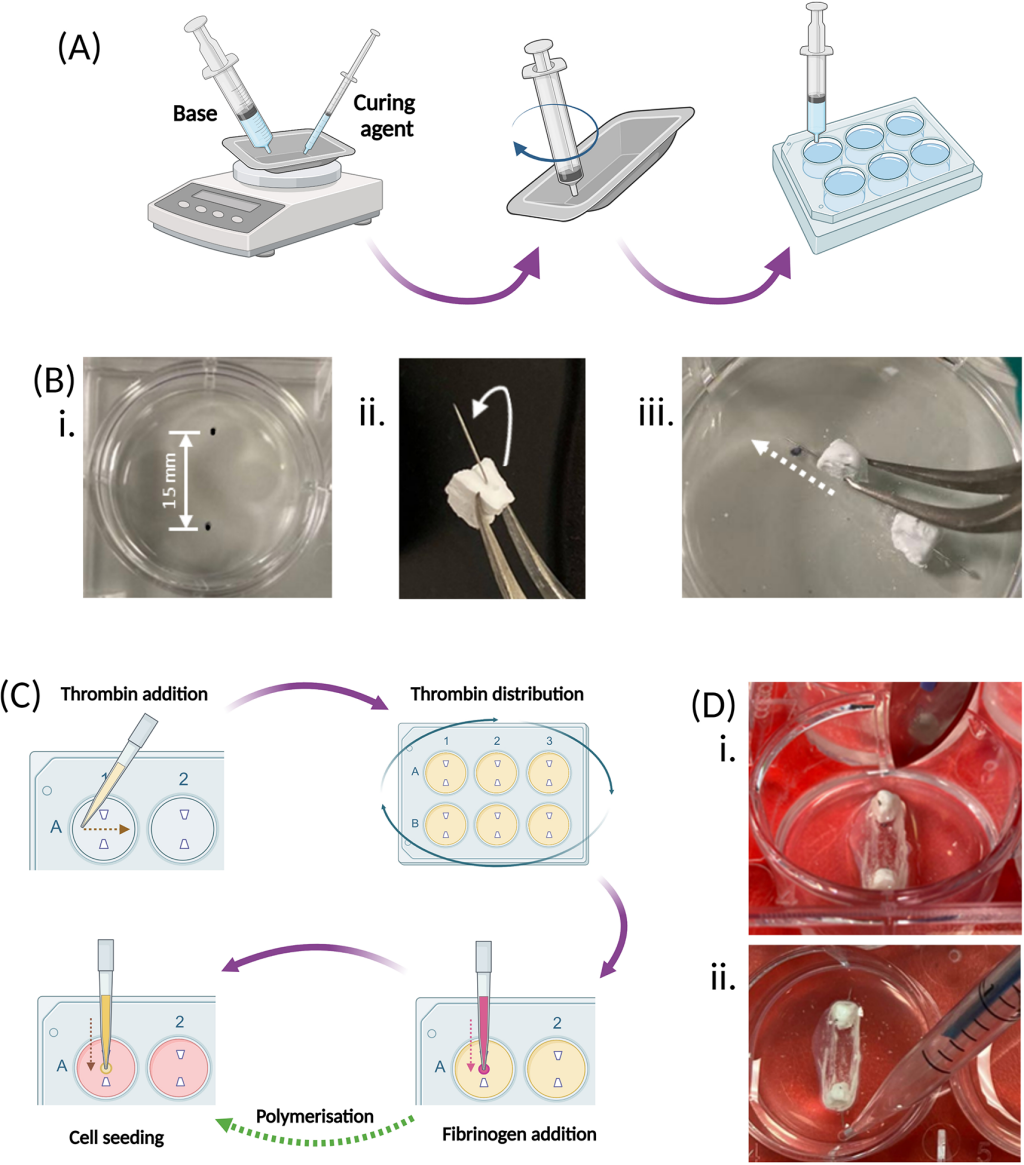
Plate and hydrogel preparation, cell seeding and construct maintenance. (A) The elastomer base, followed by the curing agent, are weighed at a 10:1 ratio into the same weighing boat, and then thoroughly mixed with the end of an empty syringe. The mixture is then transferred to an empty 6-well culture plate using this same syringe and left to set for 7 days. (B) Once the elastomer has set, 2 β-TCP cement anchors are inserted into each well of the 6-well plate. Firstly, 2 marks are made 15 mm apart on the underside of the plate for each well (i). The pins within the β-TCP anchors are bent at a slight angle, towards the long edge of the trapezoidal base using tweezers (ii), then the pins are inserted at an angle into the elastomer (white arrows), using the measured points as guide, so that the base of the anchors sit on the top surface of the elastomer (white dotted line) (iii). (C) SSBM set up involves preparing the hydrogel by addition of the thrombin across each well of the plate, and ensuring the whole surface is covered by gently mixing and tapping the sides of the plate. Addition of fibrinogen straight into the centre of each well begins polymerisation and creation of the hydrogel. Cells are seeded directly on top of the polymerised hydrogel and cultured under normal culture conditions. (D) 3 days post-cell seeding, a scalpel is run around the walls of each well to detach any fibrin from the edge of the culture well. (i). When pipetting from the well, such as during media renewal, the plate is tilted forward to pool the liquid at the bottom to ensure that the fibrin matrix is not disturbed (ii). Created with Biorender.com.

Steps:

1. Weigh out 10 ± 0.1 g of the base component of the elastomer kit into a weighing boat using a 50 mL syringe [ Figure 3.A].
2. Weigh out 1.0 ± 0.1 g of the curing component of the elastomer kit into the same weighing boat as the base component using a 1.0 mL syringe [ Figure 3.A].
3. Mix the base and curing components and slowly take up the mixture using a 10 mL syringe [ Figure 3.A].
  • *Tip – Use the end of the 10 mL syringe to mix the components.*
  • *Tip – Hold the weighing boat at an angle when taking up to allow the mixture to collect.*
  • *Tip – Continue mixing the components at regular intervals whilst taking it up.*
4. Add 1.5 mL of the mixture to each well of the plate [ Figure 3.A].
  • *Tip – If preparing more than one plate, weighing boats and syringes can be reused.*
5. Return the lids onto the plates and allow elastomer to set solid at room temperature for 7 days.
  • ***Note - Plates can be stored at room temperature after elastomer has set (no expiration has been found for using these plates).***
6. Once the elastomer has set, mark two anchor points on the underside of the plates, approximately 15 mm apart on each well [ Figure 3.B.i].
7. Bend the inserted pins towards the longer edge of the calcium phosphate anchor [ Figure 3.B.ii] and insert into the elastomer at the marked point so that the bottom of the anchor sits on the top surface of the elastomer [ Figure 3.B.iii].
  • ***Note – Plates with inserted anchors can be stored at room temperature and must be used within 6 months.***

**3. Reagent preparation for the fibrin hydrogel**

*3.1 Phosphate buffered saline (PBS) solution*

Required:

• Gibco Phosphate buffered saline (PBS) tablets (1X) (cat no.: 18912014; supplier: Fisher Scientific)
• Distilled water (d.H _2_O)

Steps:

1. Dissolve PBS tablets in d.H _2_O at a ratio of 1 tablet per 500 mL d.H _2_O.
2. Store at room temperature.

*3.2 Fibrinogen solution (20 mg/mL)*

Required:

• Fibrinogen (cat no.: F8630; supplier: Sigma-Aldrich, Gillingham, UK)
• Ham’s F-12K (Kaighn’s) Medium (F-12K medium) (cat no.: 21127-022; supplier: Fisher Scientific)
• BD Emerald disposable syringe (10 mL) (Cat no.: 15205093; supplier: Fisher Scientific)
• 0.22-μm PES (Sterile) syringe filters (cat no.: E4780-1226; supplier: Starlab, Milton Keynes, UK)
  ○ ***Note – Several PES filters might be required***

Steps:

1. Place F-12K medium in 37 °C water bath for at least 20 mins.
2. Weigh out at least 100 mg of fibrinogen ( *X*) and use the following calculation to determine volume of media ( *Y*) required:
Xmgfibrinogen/20=YmLofF−12K
3. In a cell culture hood, dilute fibrinogen in calculated volume of F-12K medium - do not vortex.
4. Place in a 37°C water bath for 1 to 2 hr, gently agitating every 30 min.
5. In a cell culture hood, filter through 0.22-μm filters with the syringe, then store at -20 °C if not using straight away.

*3.3 Aprotinin (10 mg/mL)*

Required:

• Aprotinin (cat no.: A4529-10mg; supplier: Sigma-Aldrich)
• PBS solution [Section 3.1]
• BD Emerald disposable syringe (2.0 mL) (Cat no.: 15285083; supplier: Fisher Scientific)
• 0.22-μm PES (sterile) syringe filters (cat no.: E4780-1226; supplier: Star Lab)

Steps:

1. Reconstitute entire contents of aprotinin vial using PBS solution at a ratio of 10 mg aprotinin to 1 mL PBS solution
  • ***Note – the above is for a 10 mg aprotinin vial. If purchased a different size, adjust resuspension volume accordingly to acquire 10 mg/mL***
  • *Tip – If acquired a 10 mg aprotinin vial, add 1 mL PBS solution directly into the aprotinin vial.*
2. Under sterile conditions, filter through 0.22-μm syringe filter with the syringe and prepare into 30 μL aliquots. Store at -20 °C.
  • ***Note - 1x 30 μL aliquots prepares 4x six-well plates. Avoid freeze-thaw cycles.***

*3.4 Aminohexanoic acid (200 mM)*

Required:

• Aminohexanoic acid (cat no.: 07260-10g; supplier: Sigma-Aldrich)
• PBS solution [Section 3.1]
• BD Emerald disposable syringe (5.0 mL) (Cat no.: 15295083; supplier: Fisher Scientific)
• 0.22-μm PES (sterile) syringe filters (cat no.: E4780-1226; supplier: Star Lab)

Steps:

1. Weigh out at least 50 mg aminohexanoic acid ( *X*) and use the following calculation to determine volume of PBS required ( *Y*):
Xmgaminohexanoic acid/26.2=YmLofPBS
2. In a cell culture hood, resuspend in determined volume of PBS. Vortex until completely dissolved.
3. In a cell culture hood, filter through 0.22-μm filter with the syringe and prepare into 30 μL aliquots. Store at -20 °C.
  • ***Note - 1x 30 μL aliquots prepares 4x six-well plates. Avoid freeze-thaw cycles.***

*3.5 Thrombin (200 U/mL)*

Required:

• Thrombin, bovine (cat no.: 605157 – 1KU; supplier: Sigma-Aldrich)
• Bovine serum albumin (BSA) (cat no.: A8806-5g; supplier: Sigma-Aldrich)
• Ham’s F-12K (Kaighn’s) Medium (cat no.: 21127-022; supplier: Fisher Scientific)
• 21G disposable needle (cat no.: SYR6232; supplier: SLS Scientific, Wilford, UK)
• BD Emerald disposable syringe (2.0 mL) (Cat no.: 15285083; supplier: Fisher Scientific)
• 0.22-μm PES (sterile) syringe filters (cat no.: E4780-1226; supplier: Star Lab)
  ○ ***Note – The steps for thrombin preparation below is for a single 1 KU vial. Adjust accordingly if using a different quantity.***

Steps:

1. Prepare 0.1% BSA solution by weighing out at least 6 mg BSA and use the following calculation to determine volume of F-12K medium required:
XmgofBSA=XmLofF−12Kmedium
2. Using the needle and syringe, pierce through the rubber stopper to reconstitute the entire content of the thrombin vial in 500 μL of 0.1% BSA solution
3. Remove the syringe and invert the vial until the thrombin is completely dissolved, then remove the rubber stopper and reconstitute entire contents of vial in a single 4.5mL aliquot of 0.1% BSA solution.
4. In a cell culture hood, filter the reconstituted thrombin through a 0.22-μm filter with the syringe and prepare 700 μL aliquots. Store the aliquots at -20 °C.
  • ***Note - A 700 μL aliquot is sufficient to prepare 4x six-well plates. Avoid freeze-thaw cycles.***

**4. Preparing the fibrin hydrogel and construct set up**

Required:

• Thrombin (200 U/mL)
• Aminohexanoic acid (200 mM)
• Aprotinin (10 mg/mL)
• Cell media (Non-osteogenic; refer to your cell supplier guidelines)
• Fibrinogen solution (20 mg/mL)
• Anchored plates

Steps:

1. Prior to cell seeding, spray the inside and outside of the plates with 70% ethanol and leave to dry in a sterile cell culture hood with the lids off
2. Determine the number of 6-well plates to be prepared. See Table 1 to determine volumes required for thrombin solution preparation, based on the number of 6-well plates to be prepared.
3. Thaw the appropriate number of aliquots of each of the above reagents and the fibrinogen solution and warm the media in a 37 °C water bath.
4. Once thawed, in a cell culture hood, mix the thrombin solution reagents [ Table 1] in their required quantities, based on the number of plates to be prepared and the volumes given in Table 1 below.
5. Add 500 μL of thrombin solution across the elastomer surface between the two anchors of each well of the plate(s) - do not add onto the side of the wells. Ensure the culture surface of the well is completely covered [ Figure 3.C].
  • *Tip – Swirl and tap the sides of the plate to encourage even coverage, which can be challenging due to the hydrophobic nature of the elastomer.*
6. Add 200 μL of fibrinogen solution directly onto the thrombin solution between the two anchors of each well of the plate(s) - do not add onto the side of the wells. Immediately and gently, swirl the plate to ensure complete mixing of the fibrinogen and thrombin solutions [ Figure 3.C].
7. Incubate the plates at 37 °C in an incubator for 20-30 min to allow for polymerisation. If the intention is to use the plates immediately, during this polymerisation step, isolate the cells to be used and achieve the appropriate seeding density [ Table 2].
  • ***Note - after polymerisation, plates can be stored at 2-8°C for up to a week. Preheat in an incubator at 37 °C for 30 mins prior to cell seeding.***
8. Seed 3.5 mL of cell suspension at the appropriate seeding density [ Table 2] directly onto the fibrin hydrogel between the two anchors of each well of the plate(s) - do not add onto the side of the wells. Briefly swirl the plate and incubate under normal cell culture conditions.

**Table 1.** Thrombin solution preparation. Reagents and volumes required per 6-well plate to be prepared *(Note - Volumes below account for an excess).*

Reagent	Volume (μL)
Thrombin	162.5
Aminohexanoic acid	6.5
Aprotinin	6.5
Media	3074.5

**Table 2.** Seeding density guide. Seeding density required for construct set up depends on the cell type being used. *(Note - When using a new cell type, trial different seeding densities – in general, the faster the proliferation rate, the lower the seeding density required).* Once cells are seeded, the constructs enter a growth phase, where cells proliferate and rearrange the matrix. See “expected results post-cell seeding” section for a description of expected observations in the hours/days after initial generation of SSBM constructs.

Cell line	Seeding density (cells/mL)	Osteogenic media *
Primary rat periosteal/mouse calvarial	0.1 × 10 ^6^	DMEM high glucose (Gibco), 10% FBS (Biosera), 1% PenStrep, 10 mM β-glycerophosphate, 0.1 mM ascorbic acid, 10 nM dexamethasone
MC3T3-E1	0.04 × 10 ^6^	α-MEM (Sigma), 10% FBS (Biosera), 2 mM L-Glutamine (sigma), 1% PenStrep (Sigma), 10 mM β-glycerophosphate, 50 μg/mL ascorbic acid
Primary human	0.1-0.3 × 10 ^6^ **	DMEM (Sigma), 10% FBS (Biosera), 100 Units/mL PenStrep (Sigma), 1% NEAA, 2mM β-glycerophosphate, 50 μg/mL ascorbic acid, 10 nM dexamethasone
hFOB 1.19	0.1-0.2 × 10 ^6^	DMEM/F-12 no phenol red; 10% FBS; 0.3mg/mL G418; 100 μg/mL ascorbic acid (sigma); 10-8 M menadione (Sigma)

* Suggested media recipes, user discretion should be applied.

** High variation between individual donors.

**5. Maintenance of constructs**

Required:

• Disposable sterile scalpel (cat no.: 0511; supplier: Swann-Morton, Sheffield, UK)
  ○ *No specific blade size required – blade No. 24 used here*
• PBS solution [Section 3.1] – Autoclave sterilised
• Trypsin-EDTA (2X) (cat no.: SLCC7608; supplier: Sigma-Aldrich)
  ○ ***Note – Diluted from 10X stock conc. to 2X conc. using sterile PBS solution***
• Non-osteogenic cell culture media – refer to your cell supplier guidance
• Osteogenic cell culture media – refer to your cell supplier guidance

Steps:

*3-4 days post cell seeding – fibrin detachment*

*This step aids fibrin contraction by detaching any fibrin that may have adhered to the well walls and is only required if no contraction is observed, or if part(s) of the fibrin appears ‘pinched’ by the elastomer.*

1. Slowly run a scalpel around the edges of the wells at the top surface of the elastomer or underneath the pinched area(s) [ Figure 3.D.i] to detach any fibrin that has not successfully retracted.
  • *Tip – Run the scalpel from the 12 o’clock to the 6 o’clock points in the clockwise and anti-clockwise directions – do not run the scalpel around the whole well in one direction as this risks dislodging the fibrin from the anchors*

*Media change*

1. Remove 1.5 mL of media/well and replace with 1.5 mL of non-osteogenic cell culture media [ Figure 3.D.ii]
2. Continue to renew media every 3-4 days by removing and replacing 1.5 mL media/well. Ensure each well contains a minimum media volume of 3.5 mL

*1-2 weeks post cell seeding – trypsin wash*

*This step is to prevent cell adherence and matrix formation outside of the fibrin construct*

1. Remove all media from each well and wash with 1.0 mL PBS. Discard the PBS.
2. Add 0.5 mL of 2X Trypsin-EDTA to each well and incubate at 37 °C for 3 mins. Gently tap the sides of the plate and add 1.0 mL cell culture media.
3. Remove entire volume of Trypsin-EDTA/media solution and replace with 3 mL non-osteogenic cell culture media.

2-4 weeks* post cell seeding – osteogenic incubation

Following the growth phase (see above), constructs enter an osteogenic phase where cells are encouraged to differentiate and produce new bone matrix.

* *Osteogenic incubation can begin at the discretion of the user; commonly at 4 weeks post-cell seeding, or earlier if fibrin matrix width becomes < 3 mm*

1. Remove all non-osteogenic media volume from each cell culture well and replace with osteogenic media [ Table 2].
2. Continue to culture constructs with osteogenic media, renewing every 3-4 days.
  • *Note – the total osteogenic phase duration is at the discretion of the user. A starting point and the regime used in our research group is analysis at 0-, 4-, 8- and 12-weeks of osteogenic culture (i.e., 4-, 8- and 12-weeks post seeding).*

**6. Preventing contamination**

Adhering to stringent aseptic techniques and conditions performed during normal cell culture is, in our experience adequate to prevent contamination, however as these are long-term cultures, extra precautions can be taken to reduce the risk of contamination occurring as follows:

1. Equipment decontamination; More frequent deep cleaning of incubators and cell culture hoods is recommended.
2. Designate equipment; assign equipment to only be used for SSBM culture (e.g. pipettes, pipette tips, incubators) to prevent contamination with other cell lines in use in the laboratory.
3. Sterilising of media; regularly filter media, or aliquot small amounts of media from the prepared bottle, to reduce the risk of contamination.
4. UV light; decontaminate incubators and hoods with UV light, before and or/after use.

## Results and analytical methods

### Expected results post-cell seeding

**Figure 4.**
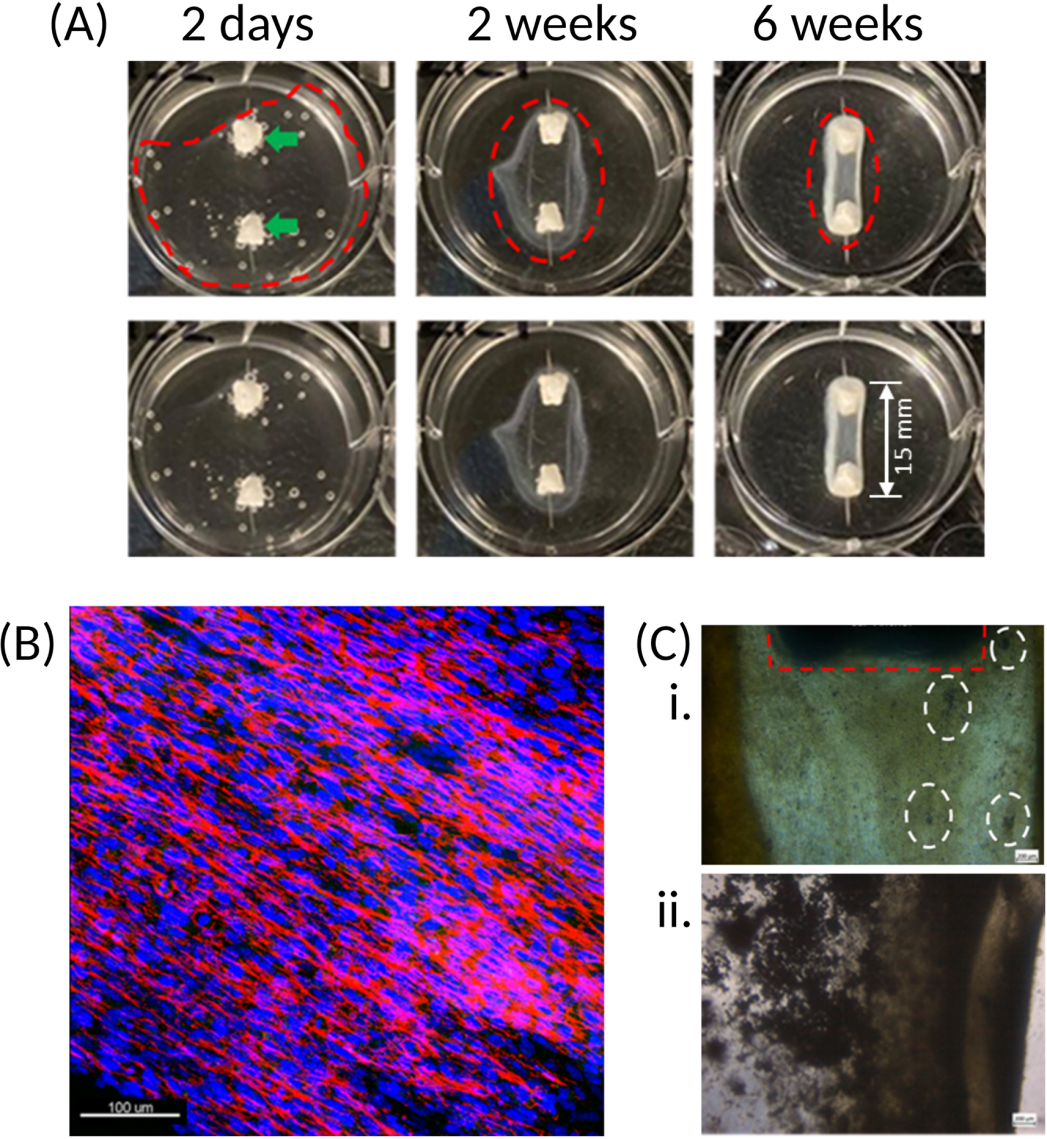
Matrix and cell organisation over time. (A) Once cells are seeded onto the fibrin hydrogel, contraction of the matrix around the two calcium phosphate anchors (green arrows) occurs. Upper and lower panels are the same images, with construct outline (dotted red line) excluded in the lower panel for clarity. Over time, the cells progressively form an organised and coordinated construct (as indicated by the narrowing of the red dotted rings). The resulting structure has a defined border-due to fibrin accumulation – which becomes stiffer and increasingly opaque following introduction of osteogenic conditions at 4 weeks. (B) Immunofluorescence staining of the cell nuclei (blue) and actin (red) shows that the cells present within the fibrin matrix align longitudinally between the two anchors, resulting in a striated configuration. This Z-stack maximum intensity projection image is of a construct cultured for 4-weeks. 20x magnification, scale bar is 100 μm. Stains: Hoechst (cat no.: 62249; supplier: Fisher Scientific) for nuclei (blue), phalloidin-488 (cat no.: A12379; supplier: Fisher Scientific) for actin (red). (C) Initially, mineral deposits are observed as dark specks (white dotted rings) of varying size within the matrix of the SSBM constructs (i). Following introduction of osteogenic media at 4 weeks, these become denser and more apparent (ii). Calcium phosphate anchor within red dotted ring. Images taken with light microscope; 4x magnification. Scale bars are 200 μm.

The first significant observation is contraction of the fibrin matrix around the anchors [ Figure 4.A.] which, depending on the cell source used, can occur in as little as 24 hours or up to 7 days post cell seeding. Cell-matrix interactions between the cell-bound integrins and Arg-Gly-Asp (RGD)-containing moieties, such as vitronectin, found within the extracellular matrix (ECM), ^24^ are essential to cell behaviour, phenotype and survival. 2D culture platforms typically adhere cells through weak electrostatic forces on one side of the cell, which encourages an unnatural flattened conformation. By seeding cells in a 3D, naturally occurring matrix, the strong affinity between these integrins and ECM components on all surfaces of the cells results in complete enrobing of the cells in the matrix, which is observed as matrix contraction. ^25^ Furthermore, with the presence of the two anchors in the well, the matrix contraction is controlled and forced into a cylindrical-like structure, whereas a disorganised spheroid will form in their absence. ^25^ Because of the cylindrical matrix, the cells themselves are organised into a parallel and striated conformation [ Figure 4B], which provides an in-built strain that further encourages cell differentiation and bone formation.

Over time, the fibrin matrix contracts into a more defined structure, which then becomes increasingly opaque upon culturing under osteogenic conditions. This opacity is due to the occurrence of osteoid deposition by the cells and its subsequent mineralisation on the matrix [ Figure 4.C].

### Analytical efficiency

**Figure 5.**
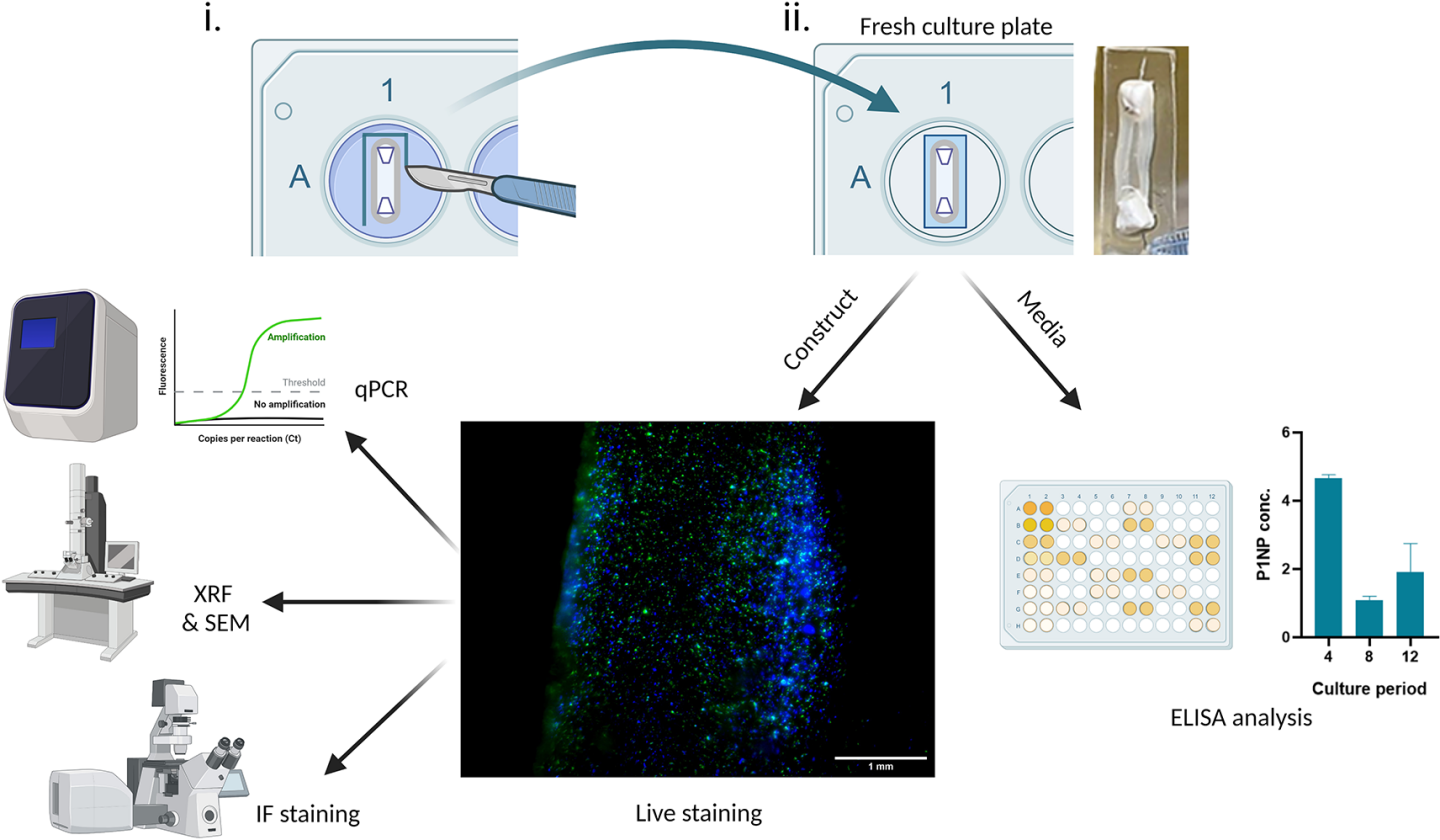
Achieving efficient analysis of SSBM cultures. (i) For analysis of exposed SSBM construct media, 3-4 days prior to the chosen analytical timepoint, intact SSBM constructs are isolated from their culture plates by cutting a strip of elastomer surrounding it, (ii) then incubated it in a fresh culture plate. At the timepoint, media can be collected and used for analysis, such as enzyme-linked immunosorbent assay (ELISA). The remaining construct can also be used for further analysis, such as live-dead imaging with ReadyProbes™ (cat no.: R37609; supplier: Invitrogen). Greater analytical efficiency can be achieved by using live-dead imaged constructs for other analytical approaches. To date, methods that have been optimised, and are under development, for SSBM construct analysis include quantitative polymerase chain reaction (RT-qPCR), x-ray fluorescence (XRF), scanning electron microscopy (SEM) and immunofluorescent (IF) staining. Created with Biorender.com.

With the correct planning, many analytical approaches can be carried out using a single construct, which demonstrates the analytical efficiency of the SSBM construct method; for example, Figure 5 shows the journey of a single construct through five separate analytical techniques. First the construct is stained intact using non-toxic ReadyProbes™ (cat no.: R37609; supplier: Invitrogen, Hertford, UK) for live-dead imaging. Subsequently, it is subjected to X-ray fluorescence (XRF) and then scanning electron microscopy (SEM). Meanwhile, the media collected from these constructs can be analysed by enzyme-linked immunosorbent assays (ELISA) at multiple timepoints throughout the construct culture period. Constructs stained with the ReadyProbes™ are also in an acceptable condition to be used for other analytical techniques, such as RT-qPCR and immunofluorescence (IF) staining.

*SSBM isolation*

The protocol above details the measures used to ensure cells attach to and remain within the fibrin matrix, and prevent cell adherence and proliferation occurring outside of the fibrin matrix – i.e. using hydrophobic Sylgard 184 silicone elastomer and regularly treating the wells with trypsin-EDTA. Although hydrophobic surfaces won’t completely prevent cell attachment (as previously demonstrated by the murine osteoblastic cell line, MC3T3), they can reduce cellular proliferation rate. ^26^ Furthermore, trypsin-EDTA is a calcium chelator, ^27^ which could affect the development of calcium-phosphate mineral, therefore it is used sparingly throughout the culture period. Given this, two separate cell populations exist within the culture dish - one outside and the other inside the SSBM construct. This is problematic for methods that involve analysing exposed media, (e.g. ELISA) as the results will reflect the activity of both cell populations, rather than just those within the SSBM construct. To overcome this problem, prior to media analysis, SSBM constructs must be isolated from their original culture plates and transferred into fresh plates. This can be achieved whilst retaining their shape integrity, and viability, by dissecting the elastomer as close as possible to the SSBM construct, with the anchors still attached, and placing it into a new 6-well dish [ Figure 5]. Following culture in the appropriate media (i.e. complete or osteogenic media) for 3-4 days, the media is collected and used for analysis immediately, or stored for analysis at a later date (refer to manufacturer’s guidance for any specific preparation steps or storage requirements for cell supernatant, prior to beginning analysis or storing) – long-term storage at -80 °C is adequate for most analysis.

### Biological analysis

*Live-dead imaging*

There are many approaches that can be used to determine cell viability based on their enzymatic and metabolic activity (e.g. MTT tetrazolium reduction, resazurin reduction and protease activity assays), or their membrane integrity (e.g. trypan blue exclusion and DNA binding dyes). However, these methods are often destructive, irreversible or cause long-term cytotoxicity. ReadyProbes™ penetrate the SSBM construct matrix and enable simple, as well as fast viability testing, without causing cell damage or cytotoxicity. Following manufacturer guidance, constructs still attached to their anchors and a strip of elastomer (as indicated above [ Figure 5]) are resuspended and incubated for 30 min (33.5°C or 37 °C, 5% CO _2_) in 3.5 mL media with 2 drops/mL of NucBlue ^®^ Live and NucGreen ^®^ Dead reagents added directly, then imaged using a fluorescence microscope with standard DAPI (excitation/emission: 360/460 nm) and FITC/GFP filters (excitation/emission: 504/523 nm).

*RNA extraction for RT-qPCR*

Reverse transcription quantitative polymerase chain reaction (RT-qPCR) is a widely used technique that enables gene expression analysis in cells and tissue samples. During osteoblast differentiation, from progenitor cells through to osteocytogenesis, there are notable and patterned changes in the expression of specific genes, which have been reviewed extensively. ^28^^–^^30^ Data from RT-qPCR can be used as an indicator of osteoblast cell maturity within the SSBM construct, as well as to provide insights into how cells are responding to particular stimuli (e.g. bone formation modulators) and culture environment etc.

**Figure 6.**
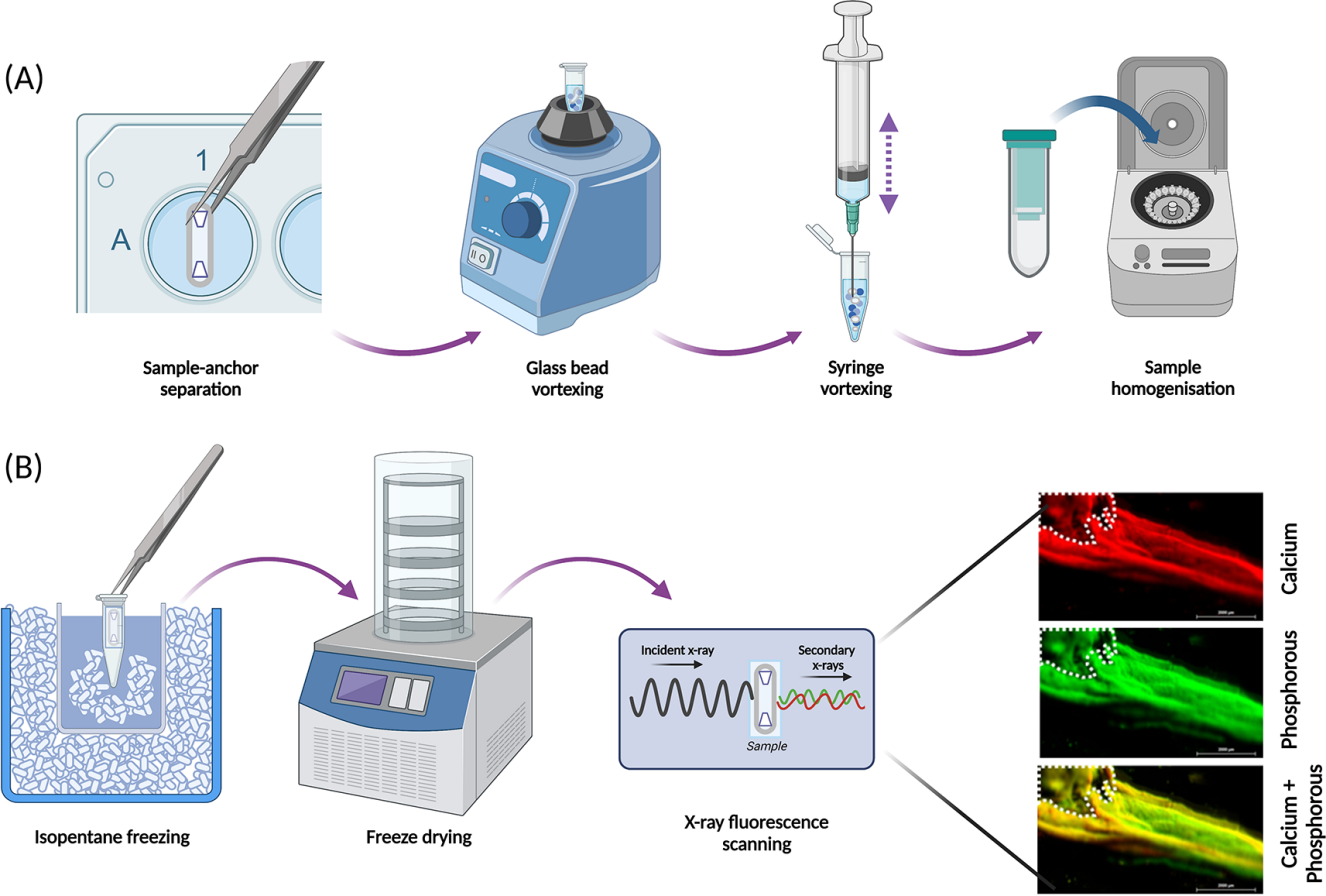
Optimised approaches for structural analysis of SSBM constructs. (A) SSBM constructs are disrupted and homogenised prior to extraction of RNA for RT-qPCR analysis, by following a process optimised for these constructs. Disruption involves vortexing samples, without the anchors, with glass beads, then by repeated aspiration using a syringe and needles. Disrupted samples are homogenised using QIAShredders (cat no.: 79656; supplier: QIAGEN), with the resultant sample ready for RNA extraction. (B) The elemental composition of the mineral present on SSBM constructs can be analysed using x-ray fluorescence (XRF). Whole constructs are first snap frozen in a dry ice bath using isopentane, immediately followed by dry freezing. XRF produces elemental map read-outs and energy spectra which can be used to locate and determine mineral distribution and maturity. The elemental maps shown here reveal co-localisation of calcium (red) and phosphorous (green), resulting in a yellow colouration, suggesting presence of a calcium phosphorous compound (yellow) i.e. bone-like mineral. Β-TCP anchors within white dotted outline. Scale bar is 2.0 mm. Created with Biorender.com.

Analysis by RT-qPCR (or RNA sequencing) relies on extraction of RNA of satisfactory quantity and quality, which is greatly influenced by the first steps in the extraction method; disruption and homogenisation of the cells and/or tissue lysates. This method has been developed and optimised for extracting good quality RNA from the SSBM constructs [ Figure 6A], using the QIAGEN Mini RNEasy ^®^ Kit (cat no.: 74104; supplier: QIAGEN, Manchester, UK).

First, the SSBM construct must be unhooked from the anchors, then placed it in a 2.0 mL eppendorf tube containing 10-15 3 mm diameter glass beads (cat no.: Z143928-1EA; supplier: Sigma-Aldrich) and 600 μL of buffer RLT containing 10% β-mercaptoethanol (v/v) (cat no.: M6250; supplier: Sigma-Aldrich). Each sample is vortexed (bench top vortex) for 1-2 minutes, followed by shredding by repeated aspiration with a syringe first using a 16G (cat no.: 11532445; supplier: Fisher Scientific) needle, then a 19G needle (cat no.: 15341537; supplier: Fisher scientific), the construct and the buffer RLT mixture is repetitively aspirated with the syringe until the construct is a fine powder in suspension. Care is needed as a vacuum is created in the presence of large pieces of construct. To reduce RNA loss, the same syringe is used between needles for the same sample; but to prevent cross contamination, a new syringe and set of needles are used for each individual SSBM construct. The resultant lysate is transferred to a QIAshredder spin column (cat no.: 79654; supplier: QIAGEN) and centrifuged (2 min; max speed in a microcentrifuge), followed by a repeat centrifugation of the resultant supernatant, with the shredder removed and collection tube capped. The supernatant is transferred by careful pipetting - to ensure no pelleted debris is taken up - to a new 1.5 mL Eppendorf and then used for RNA extraction following the steps outlined by the QIAGEN RNEasy ^®^ Mini handbook, beginning with addition of 70% ethanol to the supernatant (section “Protocol: Purification of Total RNA from Animal Tissues”, pages 51-52, as of October 2019). Typical RNA yields for hFOB 1.19 are shown in Table 3, with the purity of the RNA extracted deemed acceptable when the ratio of absorbance at 260 and 280 nm is 2.0 ± 0.15.

**Table 3.** Range of RNA yield following extraction from hFOB 1.19 SSBM constructs. Note - resultant yields will vary considerably with the culture period time and culture conditions, the cell type used to create the SSBM constructs and user experience. Values are the range obtained from successful RNA extractions performed previously (sample number, n, given for each timepoint).

Culture period post-cell seeding (post-osteogenic introduction)	RNA Concentration (ng/μL)	n
4 (0) weeks	211.8–802.7	6
8 (4) weeks	145.0–1022.8	8
12 (8) weeks	69.2–409.9	11

### Structural analysis

*Fixation*

The structure of SSBM constructs must be preserved at the elected timepoints to obtain an accurate snapshot of structural development and changes. Like bone, SSBM constructs are composite “tissues”, containing an organic and an inorganic phase, which require different methods of preservation. SSBM constructs that are to be used for structural analysis (for either the organic or inorganic phase) must first be fixed, whilst remaining attached to the anchors (as indicated above [ Figure 5]). This begins with an incubation of 4.0 mL/construct 4% formaldehyde (cat no.: HT501128; supplier: Sigma-Aldrich) for 4 hours on a shaker plate at 4.0 °C; followed by three washes with 4.0 mL/construct PBS. Constructs can then be stored in PBS at 4.0 °C for up to 2 weeks. Fixation of SSBM constructs by paraformaldehyde preserves the organic phase whilst retaining the inorganic mineral phase.

*Freeze-drying*

For structural analysis that particularly concerns the inorganic phase of the SSBM constructs, moisture must be removed from samples. Freeze-drying (lyophilisation) is the preferred method to remove water from SSBM constructs, as it maintains sample structure (both microscopically and chemically) better than alternative approaches such as oven-drying. ^31^^,^^32^

SSBM constructs must first be frozen rapidly, as slow freezing can cause crystallisation of the ice, which will distort and damage the samples. This can be achieved using liquid nitrogen immersion, however, with the SSBM constructs, this can be too vigorous and can result in damage to the fragile constructs. A more successful alternative is to freeze in an isopentane bath [ Figure 6.B]. Place a glass beaker, half-filled with isopentane and a small amount of dry ice, into a container of dry ice, ensuring the beaker is surrounded completely. SSBM constructs attached to their anchors and a strip of elastomer (as indicated above [ Figure 5]) are placed in an Eppendorf tube and submerged in the isopentane beaker for 5 minutes. Ensure that the Eppendorf is completely submerged and add additional dry ice pellets to the isopentane if the bubbling begins to slow/stop. The submersion time can be increased if the construct is not completely frozen after 5 minutes. Once frozen, constructs are stored at -80 °C, or immediately freeze-dried (Lablyo mini, York, UK) at -50 °C for at least 4 hours. Freeze-dried constructs can be stored in a sealed container at room temperature, away from moisture, for up to 6 months.

*X-ray fluorescence*

X-ray fluorescence (XRF) is an inexpensive, non-destructive and rapid analytical approach to investigate the elemental composition of samples. ^33^^–^^36^ In this case, the location and extent of calcium and phosphorous within the SSBM constructs can be assessed. Constructs are placed in an XRF (M4 Tornado, Bruker Nano Gmbh, Germany) under a 20 mBar vacuum. After adjusting the focus on the construct, the region of interest (ROI) is outlined and then scanned with the X-ray (50 kV, 400 uA) at 1000 spots/10 mm (spot size = 25 μm), 5 ms/pixel exposure time.

By overlaying the calcium and phosphorous elemental maps [ Figure 6.B], the resultant yellow colouration indicates co-localisation of the two elements, which strongly suggests the presence of calcium-phosphate mineral compounds. The resultant maps and spectra provide further information regarding the distribution and maturity of the mineral present through the varying degrees of colour intensity visualised on the maps, which are determined by the size of each element’s unique energy signal. Regarding the SSBM constructs, mineral distribution and maturation appears heterogeneous as found in *in vivo* bone tissue. ^37^

### Immunofluorescent staining

**Figure 7.**
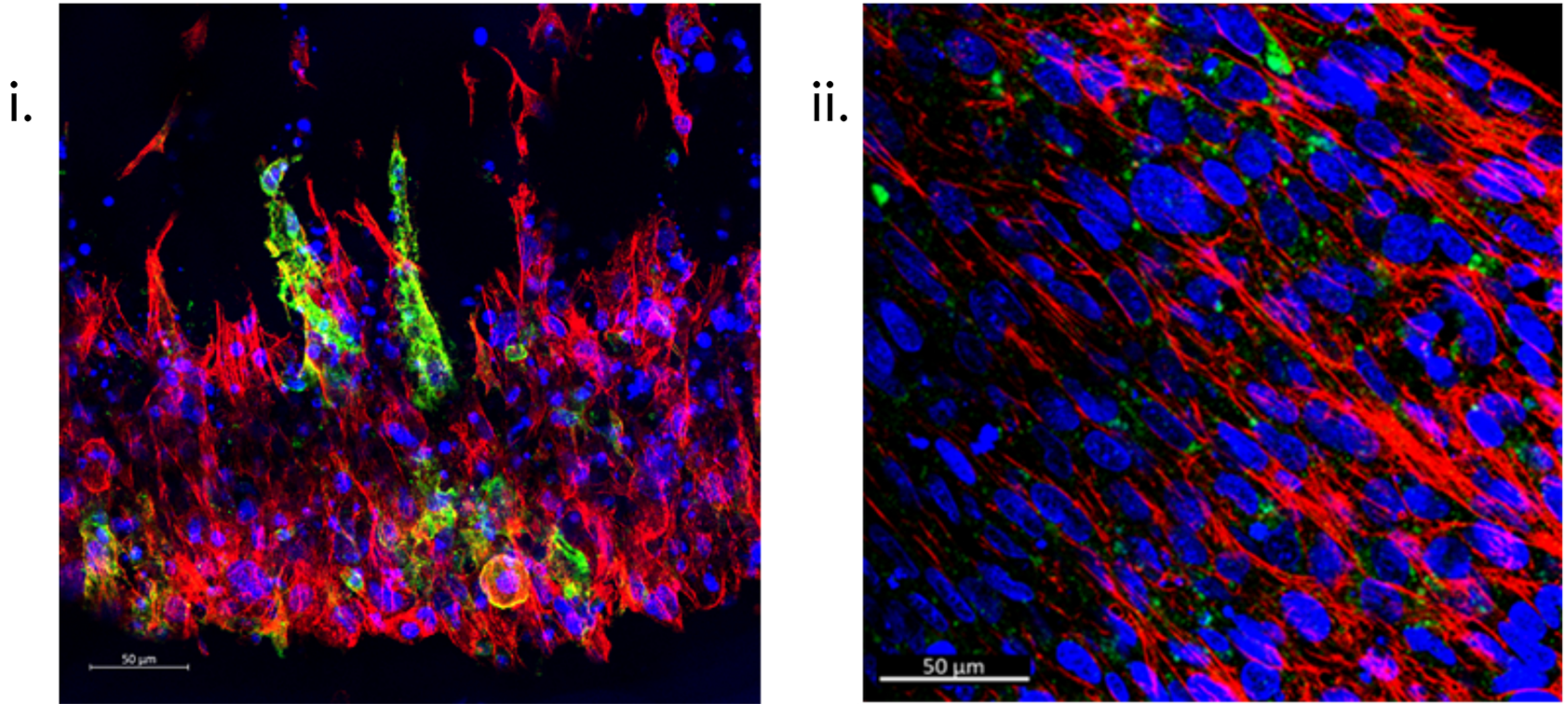
Immunofluorescent staining of SSBM constructs is achievable once sectioned or whole. Following adapted protocols for an established gelatin-based embedding, sectioning, and immunofluorescent staining protocol ^37^ (i) and one general protocol optimised for sectioned collagen-based matrices (ii), respectively, resultant 50 μm sections (i) and whole intact (ii) SSBM constructs are used to examine specific bone cell markers e.g., podoplanin (green), within the SSBM constructs, as well as an overview of cell presence and conformation. Left to right: Images above are z-stack maximum intensity projections of cultured SSBM constructs, using phalloidin for actin staining (red) and hoechst nuclei staining (blue). Scale bars are 50 μm.

Immunofluorescence (IF) staining techniques offer the capability to visualise the structural and biological characteristics of samples with high resolution and accuracy. The ability to section samples [ Figure 7.A.i] and the vast range of fluorescent secondary antibodies available means a single sample can be analysed for numerous structural and biological markers of interest, thus furthering analytical efficiency. Furthermore, SSBM constructs can also be IF stained intact, enabling a gross overview of the cellular orientation, as well as the expression of markers of interest in relation to their location within the construct within the construct [ Figure 4.B, Figure 7.A.ii].

Prior to staining, constructs must be fixed and then unhooked from their anchors – the constructs tend to retain their shape after unhooking once they have been fixed, as well as with increasing maturity (i.e. older constructs are stiffer).

*IF Staining: Sectioned constructs*

The method developed by Kusumbe *et al*., 2015 ^38^ reliably allows the preparation of bone tissue for high-resolution IF images without compromising tissue structure or proteins. Due to the delicate and bone-like nature of the SSBM constructs, this method is also ideal for their IF staining.

The method begins with sample fixation steps, which have been adapted and described above for the SSBM constructs (refer to *'structural analysis' above*), followed by decalcification and cryoprotection steps in preparation for embedding. Unlike traditional methods of embedding (e.g. paraffin embedding), this method uses gelatin-based embedding, with polyvinylpyrrolidone (PVP) and sucrose cryoprotection, as it has been found to prevent bone disintegration and allows for thicker sectioning. However, due to the fragility of the SSBM constructs in comparison to bone (i.e. a thinner composite tissue containing collagen and fibrin), the incubation conditions required for embedding are altered from 45 mins at 60 °C, to 90 mins at 45 °C. Once embedded and frozen, the protocol ^38^ outlines the steps for sectioning the resultant tissue blocks. In the case of the SSBM constructs, 50 μm sections are recommended [ Figure 7.A.i]. Finally, the method outlines the IF staining procedure. Antibody incubation durations, dilutions, and fluorophore panels are subject to the researcher’s discretion. Figure 7.A.i shows SSBM constructs stained for podoplanin using an anti-human podoplanin rabbit monoclonal antibody (1:200) (cat no.: D9D7; supplier: Cell Signaling Technology, USA), followed by an Alexa Fluor ^®^ 647 AffiniPure donkey anti-rabbit IgG secondary (1:200) (cat no.: 711-605-152; supplier: Jackson ImmunoResearch, Ely, UK). Along with hoechst (1:400) (cat no.: 62249; supplier: Fisher Scientific) for nuclei and phalloidin-488 (1:100) (cat no.: A12379; supplier: Fisher Scientific) for actin. Once stained, the SSBM construct sections are treated with ProLong™ Diamond Antifade Mountant (cat no.: P36961; supplier: Fisher Scientific) then coverslipped.

*IF Staining: Whole constructs*

A general IF staining protocol developed for collagen-based matrices, that have been paraffin-embedded and sectioned, has been developed in our laboratories and forms the basis of the method that is used for staining SSBM constructs whole and intact [ Figure 7.A.ii].

SSBM constructs that have been unhooked from their anchors, are decalcified and cryoprotected as described by Kusumbe *et al.,* 2015. ^38^ In a fresh 6-well plate, SSBM constructs are treated with blocking buffer [ Table 4] for 1 hour at room temperature (RT) – ensuring the constructs are completely submerged (>3.0 mL solution per construct) – then washed twice with PBS. Primary antibody is diluted accordingly using antibody buffer [ Table 4] and >3.0 mL solution per construct added to each well, then incubated for 2 hours at RT. Constructs are washed 3 times with PBS and treated with the secondary antibody, as well as hoechst (1:400) and phalloidin (1:100), diluted accordingly in antibody buffer [ Table 4], at >3.0 mL solution per construct for 2 hours at RT and covered. Finally, the SSBM constructs are washed 3 times with PBS and are immediately imaged on a confocal microscope, not in solution, on a glass-bottomed (170±5 μm thickness) imaging dish (cat no.: 80137; supplier: Ibidi, Germany).

**Table 4.** Reagent recipes required for whole construct IF staining.

Reagent	Final concentration	Cat no.	Supplier
**Blocking buffer**			
PBS	Diluent	18912014	Fisher Scientific
Triton X	0.1%	X100 5ML	Sigma-Aldrich
BSA	30 mg/mL	A8806-5g	Sigma-Aldrich
**Antibody buffer**			
PBS	Diluent	18912014	Fisher Scientific
Tween 20	0.5%	P1379 25ML	Sigma-Aldrich
BSA	30 mg/mL	A8806-5g	Sigma-Aldrich

### Troubleshooting

**Table 5.** Troubleshooting table.

Problem	Possible reasons	Solutions
Fibrin not contracting	- Cell seeding density too low - Cell viability too low - Fibrinogen batch-batch variation	Discard constructs that have not contracted and attempt the following solutions for subsequent set ups: - Increase cell seeding density – varies between species and origin - Ensure cell viability is >85% prior to seeding - Order new fibrinogen with a different lot number (available on fibrinogen vial)
RNA too low	- Construct not completely disrupted - Construct viability/metabolic activity too low	- Carry out cDNA reverse transcriptase with low RNA sample, followed by cDNA amplification - Vortex and aspirate subsequent constructs for longer during disruption and aspiration steps of RNA extraction
Dry freezing snapping constructs	- Constructs not completely frozen prior to freeze drying	- Submerge subsequent constructs in isopentane bath for >5 min - Try a different snap freezing method (e.g. liquid nitrogen) - Try oven drying methods
Cement hardening too quickly	- Orthophosphoric acid/equipment not cold enough - Equipment contains traces of solidified cement - Volume of orthophosphoric acid too low	- Ensure acid is stored at 4 °C and is kept cool - Set up workstation [ Figure 2.C.i] at least 30 mins prior to beginning - Ensure all equipment being used is clean (i.e. fresh weighing boats, clean spatulas etc.) - Adjust acid volume by increments/reductions of 100 μL until obtaining the desired consistency

## Conclusion

This detailed step-by-step protocol for the preparation, production, and maintenance of a novel 3D *in vitro* bone model, and the adapted analytical methods described, can accommodate various osteoblastic cell sources and species. Importantly, we have now demonstrated the utility of this model using hFOB 1.19 cells (as shown here), and previously with primary rodent osteoblasts, ^19^^,^^20^ as well as with primary human osteoblasts (data not shown). This inherent flexibility in cell source, and the minimal requirement for specialist equipment/reagents (beyond those available in most well-resourced molecular biology laboratories) widens the potential applications and accessibility for this model. The use of cell lines or, where available, primary human cells rather than primary cells from other vertebrate species, should be used to reduce the requirement for animals as the osteoblast source.

The humanisation of the method using the hFOB cell line has reduced our requirement for *ex vivo* samples considerably. We estimate that the work conducted over the past two years in our laboratory (during which time, a total of >280 hFOB-containing constructs have been produced) would have required tissue from >140 rats using the previous *ex vivo* iteration of the method. ^19^ Within the UK, there are at least 6 research groups working on bone modelling currently using animal-derived primary tissue models. Considering a similar level of animal use to our laboratory, we estimate that the development of our humanised *in vitro* model for the long-term study of bone has the potential to replace over 840 rodents per year for similar research across the UK alone. With the development potential and adaptability of the model to study normal development, as well as bone diseases and novel therapies, the replacement potential for this *in vitro* model is significant.

We anticipate that this assay could be readily adapted to incorporate mixed cell populations to study cell-cell interactions. For example, co-culture with osteoclasts to study bone remodelling or with malignant cells to study bone metastasis. Whilst acknowledging that such a culture cannot recapitulate the bone microenvironment in its entirety, assessment of the effects of candidate therapeutic compounds on construct mineralisation rate and osteoblast differentiation and function will allow screening for efficacy prior to *in vivo* studies. Such uptake of this method at the discovery stage of research, particularly with the capacity to utilise human and patient cells, will lead to a reduced requirement for *in vivo* and *ex vivo* platforms, therefore further replacing the need for the large number of laboratory animals currently required, and improve the translatability of preclinical studies.

## Data Availability

Zenodo: A detailed methodology for the long-term in vitro culture and analysis of three-dimensional, self-structuring bone models generated from cell lines or primary osteoblastic cell populations. https://doi.org/10.5281/zenodo.7620806 ^39^ This project contains the RNA yields data and original image files. Zenodo: A detailed methodology for the long-term in vitro culture and analysis of three-dimensional, self-structuring bone models generated from cell lines or primary osteoblastic cell populations. https://doi.org/10.5281/zenodo.7620806 ^39^ This project contains the following extended data:
- 6-well 20 x anchors Mold Frame.gcode - 6-well 20 x anchors.3mf Data are available under the terms of the Creative Commons Attribution 4.0 International license (CC-BY 4.0).
