# Supplementary material for: A detailed methodology for a three-dimensional, self-structuring bone model that supports the differentiation of osteoblasts towards osteocytes and the production of a complex collagen-rich mineralised matrix
Source: F1000Res. 2024 Jul 12;12:357. Originally published 2023 Mar 31. [Version 3] doi: 10.12688/f1000research.130779.3 (PMC11109547; doi:10.12688/f1000research.130779.3)
Supplement: Peer review history

# Reviewer report: Reviewer response for version 3

**DOI:** 10.5256/f1000research.168944.r302309

**Published:** 2024-07-26

**Authors:** Michelle A. Lawson

**Affiliations:** 1 The Mellanby Centre for Musculoskeletal Research, Department of Oncology and Metabolism, The University of Sheffield, Sheffield, UK

All comments have been addressed.

Is the rationale for developing the new method (or application) clearly explained?

Yes

Are the 3Rs implications of the work described accurately?

Partly

Is the description of the method technically sound?

Yes

Are the conclusions about the method and its performance adequately supported by the findings presented in the article?

Partly

If any results are presented, are all the source data underlying the results available to ensure full reproducibility?

Partly

Are a suitable application and appropriate end-users identified?

Yes

Are sufficient details provided to allow replication of the method development and its use by others?

Yes

Reviewer Expertise:

Bone and Cancer Biology

I confirm that I have read this submission and believe that I have an appropriate level of expertise to confirm that it is of an acceptable scientific standard.

# Reviewer report: Reviewer response for version 3

**DOI:** 10.5256/f1000research.168944.r302312

**Published:** 2024-07-19

**Authors:** Holger Jahr

**Affiliations:** 1 RWTH Aachen University, Aachen, Germany

I am very pleased with the revision and would like to congratulate the team of authors to their very nice contribution to this field.

Is the rationale for developing the new method (or application) clearly explained?

Yes

Are the 3Rs implications of the work described accurately?

No

Is the description of the method technically sound?

Yes

Are the conclusions about the method and its performance adequately supported by the findings presented in the article?

Partly

If any results are presented, are all the source data underlying the results available to ensure full reproducibility?

Yes

Are a suitable application and appropriate end-users identified?

Yes

Are sufficient details provided to allow replication of the method development and its use by others?

Yes

Reviewer Expertise:

bone regeneration and biomineralization

I confirm that I have read this submission and believe that I have an appropriate level of expertise to confirm that it is of an acceptable scientific standard.

# Reviewer report: Reviewer response for version 2

**DOI:** 10.5256/f1000research.165828.r274846

**Published:** 2024-05-14

**Authors:** Jesus Delgado-Calle, Hayley Sabol

**Affiliations:** 1 University of Arkansas for Medical Sciences, Little Rock, USA

The authors have addressed my concerns in this revised submission.

Is the rationale for developing the new method (or application) clearly explained?

Yes

Are the 3Rs implications of the work described accurately?

Partly

Is the description of the method technically sound?

Yes

Are the conclusions about the method and its performance adequately supported by the findings presented in the article?

Partly

If any results are presented, are all the source data underlying the results available to ensure full reproducibility?

Yes

Are a suitable application and appropriate end-users identified?

Yes

Are sufficient details provided to allow replication of the method development and its use by others?

Yes

Reviewer Expertise:

osteocyte, bone biology, cancer.

We confirm that we have read this submission and believe that we have an appropriate level of expertise to confirm that it is of an acceptable scientific standard.

# Reviewer report: Reviewer response for version 1

**DOI:** 10.5256/f1000research.143559.r169248

**Published:** 2024-05-13

**Authors:** Michelle A. Lawson

**Affiliations:** 1 The Mellanby Centre for Musculoskeletal Research, Department of Oncology and Metabolism, The University of Sheffield, Sheffield, UK

Finlay *et al. *describe a method to produce a 3D, self-structuring bone model that uses a biocompatible scaffold to support the differentiation of osteoblasts to osteocytes and the production of a complex collagen-rich mineralised matrix using hFOB 1.19 cells.

 Regarding the title, since previous bone models have limited osteocyte culture within them, should this key component not be highlighted in the title? For example, would this not be more suitable title “A three-dimensional, self-structuring bone model that uses a biocompatible scaffold to support the differentiation of osteoblasts to osteocytes and the production of a complex collagen-rich mineralised matrix”?

 In the abstract and introduction, it’s mentioned that the SSBM method can be readily adapted to accommodate osteoblasts derived from a number of different species, from primary osteoblasts isolated from vertebrate bone samples or from cell lines, but you don’t say which until p13 Table 2. These should be mentioned much earlier either in the abstract and/or the introduction. It’s also not clear if all the data shown is using the hFoB 1.19 cells or other cells too.

 The introduction is clear and describes the limitations of previous bone models and ethical issues of using animal models.

 The methods describe very detailed, point by point steps with figures on how to set up each of the 5 aspects of the model, including top tips in each step and cell seeding densities depending on species and cell type. However, for the 1 and 2 weeks post cell seeding trypsin wash (p14) it is less clear why the cells attached to the fibrin matrix will not also be detached by the trypsin. Is it because they are already enrobed in the matrix by then?

 The resulting scaffolds and analysis techniques are also described in a lot of detail along with a troubleshooting table. The culture of primary human osteoblasts is also mentioned but the paper provides no evidence of their use in this novel 3D *in vitro *bone model. Although not surprisingly patient primary cells will vary between samples in these models, so to demonstrate the model preparation, production, and maintenance the hFoB 1.19 cells are less variable, however, it would be good to see some data on the use of primary patient cells in the SSBM since the authors have stated they can be used.

**Minor points:**

- Figure 1 would be better placed in the methods section rather than the introduction.
- In the keywords “in vitro” should be written in italics.
- p5 in the methods text B-TCP needs to be written out in full.
- p12 in this calculation (X mg of BSA = X mL of F - 12K medium) F-12K is no written correctly please amend.
- P 15 Figure 4, what are the actual cells seeded onto the fibrin hydrogel in these images?
- P15 Figure 4Cii says “Calcium phosphate anchor within red dotted ring”, but is should say red dotted area.
- P19 Figure 7 (great IF images BTW) but what are the cells used in the SSBM constructs?
- Table 5 is not mentioned in the main text.

Is the rationale for developing the new method (or application) clearly explained?

Yes

Are the 3Rs implications of the work described accurately?

Partly

Is the description of the method technically sound?

Yes

Are the conclusions about the method and its performance adequately supported by the findings presented in the article?

Partly

If any results are presented, are all the source data underlying the results available to ensure full reproducibility?

Partly

Are a suitable application and appropriate end-users identified?

Yes

Are sufficient details provided to allow replication of the method development and its use by others?

Yes

Reviewer Expertise:

Myeloma-induced bone disease

I confirm that I have read this submission and believe that I have an appropriate level of expertise to confirm that it is of an acceptable scientific standard, however I have significant reservations, as outlined above.

# Response

**Published:** 2024-07-08

**Authors:** Amy Naylor

**Affiliations:** University of Birmingham, UK, UK

**Reviewer 4**

 Finlay *et al. *describe a method to produce a 3D, self-structuring bone model that uses a biocompatible scaffold to support the differentiation of osteoblasts to osteocytes and the production of a complex collagen-rich mineralised matrix using hFOB 1.19 cells.

 Regarding the title, since previous bone models have limited osteocyte culture within them, should this key component not be highlighted in the title? For example, would this not be more suitable title “A three-dimensional, self-structuring bone model that uses a biocompatible scaffold to support the differentiation of osteoblasts to osteocytes and the production of a complex collagen-rich mineralised matrix”?

*Response: We agree, and have now amended the title to:*

*“A detailed methodology for a three-dimensional, self-structuring bone model that supports the differentiation of osteoblasts towards osteocytes and the production of a complex collagen-rich mineralised matrix”*

 In the abstract and introduction, it’s mentioned that the SSBM method can be readily adapted to accommodate osteoblasts derived from a number of different species, from primary osteoblasts isolated from vertebrate bone samples or from cell lines, but you don’t say which until p13 Table 2. These should be mentioned much earlier either in the abstract and/or the introduction. It’s also not clear if all the data shown is using the hFoB 1.19 cells or other cells too.

*Response: We have found that this method supports osteoblasts from all species and sources that we have trialled. We use hFOB1.19 throughout the manuscript for simplicity and have noted this now in the abstract to ensure clarity for the reader.*

*Abstract:*

*“Osteoblastic cells (hFOB1.19) are seeded on a fibrin hydrogel, cast between two beta-tricalcium phosphate anchors.”*

*In addition, we state at the beginning of the methods section which cell source has been used throughout this manuscript. We have now added an additional reference to table 2, so that the reader is directed to this information earlier:*

*Paragraph 2, Methods:*

*“The figures included here were generated using the human osteoblastic cell line, hFOB 1.19 (RRID: CVCL_3708) – selected based on their homogenous human origin, as well their stable karyotype, ^21^ but the SSBM method can be readily adapted to accommodate osteoblasts derived from a number of different species, from primary osteoblasts isolated from vertebrate bone samples or from cell lines. We have used primary human osteoblasts and the human osteoblast cell line, hFOB 1.19, ^22^ with similar success (data not shown – recommended seeding densities are given in table 2).”*

 The introduction is clear and describes the limitations of previous bone models and ethical issues of using animal models.

 The methods describe very detailed, point by point steps with figures on how to set up each of the 5 aspects of the model, including top tips in each step and cell seeding densities depending on species and cell type. However, for the 1 and 2 weeks post cell seeding trypsin wash (p14) it is less clear why the cells attached to the fibrin matrix will not also be detached by the trypsin. Is it because they are already enrobed in the matrix by then?

*Response: The reviewer is right to note that contact with trypsin is likely to affect the attachment of all cells to their matrix. Fortunately, the SSBM constructs sit proud of the tissue culture plastic by virtue of their attachment to the anchors. By using a relatively small volume of trypsin solution (0.5 ml) compared to the usual volume of 3.5 ml, detachment of the tissue culture plastic-adherent cells without much contact with the construct itself is possible:*

*“2. Add 0.5 mL of 2X Trypsin-EDTA to each well and incubate at 37 °C for 3 mins. Gently tap the sides of the plate and add 1.0 mL cell culture media”*

*It is reasonable to assume that some detachment of cells on the underside of the construct may occur, however given that this trypsinisation process is performed only infrequently and only during the early stages (first 4 weeks only) of SSBM formation, this does not have long term implications for the analysis timepoints (8 and 12 weeks).*

 The resulting scaffolds and analysis techniques are also described in a lot of detail along with a troubleshooting table. The culture of primary human osteoblasts is also mentioned but the paper provides no evidence of their use in this novel 3D *in vitro *bone model. Although not surprisingly patient primary cells will vary between samples in these models, so to demonstrate the model preparation, production, and maintenance the hFoB 1.19 cells are less variable, however, it would be good to see some data on the use of primary patient cells in the SSBM since the authors have stated they can be used.

*Response: We agree with the reviewer that detailed assessment of the behaviour of different cell types within the SSBM would provide interesting data, however such characterisation is beyond the scope of this paper, which simply seeks to provide a detailed methodology to enable other groups to use and adapt the model for their research interests. We trust that the seeding densities provided for the cell types that we have trialled (table 2) will be useful in supporting researchers in establishing the model in their labs with their cell type of interest.*

**Minor points:**

- Figure 1 would be better placed in the methods section rather than the introduction.

*Response: Whilst we can see the logic in the reviewer’s statement, we first reference figure 1 in the introduction, therefore we have decided to leave it where it is in the manuscript.*


*  *

- In the keywords “in vitro” should be written in italics.

*Response: Thank you for this observation – we have made the change.*

  

- p5 in the methods text B-TCP needs to be written out in full.

*Response: Agreed. We have now made this addition.* 

- p12 in this calculation (X mg of BSA = X mL of F - 12K medium) F-12K is no written correctly please amend.

*Response: Now corrected.*

  

- P 15 Figure 4, what are the actual cells seeded onto the fibrin hydrogel in these images?

*Response: We have now referred to the use of “hFOB1.19” to all figures as appropriate.*


*  *

- P15 Figure 4Cii says “Calcium phosphate anchor within red dotted ring”, but is should say red dotted area.

*Response: Now amended.*

  

- P19 Figure 7 (great IF images BTW) but what are the cells used in the SSBM constructs?

*Response: Thank you! Now dealt with as above – hFOB1.1.9 added to all relevant figures.*

  

- Table 5 is not mentioned in the main text.

*Response: Thank you for spotting this. Underneath the title “Troubleshooting” we have now added the following sentence:*

*“Potential challenges experienced during SSBM generation are describe in Table 5, along with their possible causes and solutions.”*

# Reviewer report: Reviewer response for version 1

**DOI:** 10.5256/f1000research.143559.r176191

**Published:** 2024-05-08

**Authors:** Sabrina Ehnert

**Affiliations:** 1 University of Tübingen, Tübingen, Germany

The manuscript is well written and the methodology is provided in sufficient detail - except for two points:

1. How often can/should the Trypsin/EDTA treatment be done? This is a bit unclear in the description.
2. Centrifugation speed should be given in G (not max. centrifugation speed - e.g. ENA isolation).

The conclusion is a bit ambigious as using, for example, osteosarcoma cells or including osteoclast like cells may not be easily done as these cells have strong proteolytic activity and thus may dissolve the constructs.

Is the rationale for developing the new method (or application) clearly explained?

Yes

Are the 3Rs implications of the work described accurately?

Partly

Is the description of the method technically sound?

Yes

Are the conclusions about the method and its performance adequately supported by the findings presented in the article?

Partly

If any results are presented, are all the source data underlying the results available to ensure full reproducibility?

Yes

Are a suitable application and appropriate end-users identified?

Yes

Are sufficient details provided to allow replication of the method development and its use by others?

Yes

Reviewer Expertise:

Bone models and bone metabolism in vitro

I confirm that I have read this submission and believe that I have an appropriate level of expertise to confirm that it is of an acceptable scientific standard, however I have significant reservations, as outlined above.

# Response

**Published:** 2024-07-08

**Authors:** Amy Naylor

**Affiliations:** University of Birmingham, UK, UK

**Reviewer 3:**

 The manuscript is well written and the methodology is provided in sufficient detail - except for two points:

1. How often can/should the Trypsin/EDTA treatment be done? This is a bit unclear in the description.
***Response:** We thank you for highlighting this missing information and now added the following to this section (5. Maintenance of constructs):*
*“A trypsin wash is recommended to be performed at least 1 week after cell seeding, or once contraction of the fibrin has been established. Repeat the trypsin wash 2 weeks later, however, do not perform trypsin washes once osteogenic conditions have been introduced as this will impact mineralisation.”*
2. Centrifugation speed should be given in G (not max. centrifugation speed - e.g. ENA isolation).
***Response:** We agree, thank you for noting this omission – it has now been rectified:*
*”(2 min;  13 000 G) using a microcentrifuge”*

The conclusion is a bit ambigious as using, for example, osteosarcoma cells or including osteoclast like cells may not be easily done as these cells have strong proteolytic activity and thus may dissolve the constructs.

***Response:** In our opinion, the addition of additional cell types to the constructs will provide valuable insight into their activity in the context of a bone organotypic matrix and mature osteoblasts, indeed this is something that we, and our collaborators, are currently exploring. We recognise that there may be challenges in the development of the assay in this direction and have therefore softened the original sentence to reflect this:*

**

*“There is potential for this assay to be adapted to incorporate mixed cell populations to study cell-cell interactions and we encourage further development along these lines”.*

# Reviewer report: Reviewer response for version 1

**DOI:** 10.5256/f1000research.143559.r189268

**Published:** 2024-03-21

**Authors:** Holger Jahr

**Affiliations:** 1 RWTH Aachen University, Aachen, Germany

The present paper describes an *in vitro *culture method for the long-term

 analysis of 3D bone models from osteoblastic cells or cell lines. The manuscript is logically structured and well written. Despite its complexity, it leaves me with rather few questions or suggestions for improvement, respectively.

  

1. Per convention, extracted RNA is used in RT-qPCR, not qPCR. This should be corrected. Was the 260/230 nm ratio determined, too?

 

1. During plate preparation (#2), each well is pre-filled with 1.5 mL Sylgard 184 silicone elastomer solution. Subsequently, the fibrin hydrogel is prepared (#4) by adding 0.2 mL of fibrinogen solution (20 mg/mL) to 0.5 mL of thrombin solution “across” the elastomer surface, is the final concentration 8 mg/mL? Does the mixture diffuse into the elastomer to potentially alter the final concentration?

 

1. Later, 3.5 mL of cell suspension (about 525,000 cells) are seeded onto the fibrin hydrogel. Did you try using RNeasy Micro kit, too?

 

1. I am not convinced that Fig. 7 suggests the presence of osteocytes in the tissue through a single podoplanin-positive staining in the absence of controls as this protein is also expressed on other cell types. IF staining should include other accepted bone-specific or bone maturation makers. This could be easily checked and should be considered.

 

1. Importantly, while using XRF is a nice idea, demonstrating calcium and phosphorous within the SSBM constructs does not make this a “bone model”. To this end, a major limitation of the present methodology is that no biomineralization has been shown, yet, while the title suggests a “bone model” was developed. Stating that “mineral distribution and maturation appears heterogeneous as found in in vivo bone tissue” does not really convince me. Please provide further evidence.

Is the rationale for developing the new method (or application) clearly explained?

Yes

Are the 3Rs implications of the work described accurately?

No

Is the description of the method technically sound?

Yes

Are the conclusions about the method and its performance adequately supported by the findings presented in the article?

Partly

If any results are presented, are all the source data underlying the results available to ensure full reproducibility?

Yes

Are a suitable application and appropriate end-users identified?

Yes

Are sufficient details provided to allow replication of the method development and its use by others?

Yes

Reviewer Expertise:

3D models, bone regeneration, molecular biology

I confirm that I have read this submission and believe that I have an appropriate level of expertise to confirm that it is of an acceptable scientific standard, however I have significant reservations, as outlined above.

# Response

**Published:** 2024-04-29

**Authors:** Amy Naylor

**Affiliations:** University of Birmingham, UK, UK

- *Per convention, extracted RNA is used in RT-qPCR, not qPCR. This should be corrected. Was the 260/230 nm ratio determined, too? *

**Author Response: **Thank you for this comment. We confirm that we did indeed perform reverse transcription PCR, therefore we have amended the text to RT-qPCR, as suggested. 260/230 ratios were not collected because no Trizol or phenol were used during sample isolation. The 260/280 ratio was collected to give an indication of RNA quality and to detect other potential contaminants. In all cases we found this ratio to be close to 2 (a tolerance of +0.15 was considered acceptable), demonstrating reasonable purity.

  

- *During plate preparation (#2), each well is pre-filled with 1.5 mL Sylgard 184 silicone elastomer solution. Subsequently, the fibrin hydrogel is prepared (#4) by adding 0.2 mL of fibrinogen solution (20 mg/mL) to 0.5 mL of thrombin solution “across” the elastomer surface, is the final concentration 8 mg/mL? Does the mixture diffuse into the elastomer to potentially alter the final concentration? *

**Author Response: **There is a period between addition of elastomer and addition of fibrin mixture where the elastomer is left to set and solidify, therefore diffusion is not expected to occur between the fibrin matrix and the elastomer base. This is described in section 2, “plate preparation”, step 5 as follows: “Return the lids onto the plates and allow elastomer to set at room temperature for 7 days.” We have now added the word “solid” to make this clearer for the reader such that the sentence now reads: “Return the lids onto the plates and allow elastomer to set solid at room temperature for 7 days.”

  

- *Later, 3.5 mL of cell suspension (about 525,000 cells) are seeded onto the fibrin hydrogel. Did you try using RNeasy Micro kit, too?*

**Author Response: **The average weight of the constructs is approximately 50 mg, however, because they are formed from a hydrogel, this weight can vary dependent on the degree of water absorbed by/contained within it. The Qiagen product information ( https://www.qiagen.com/us/products/discovery-and-translational-research/dna-rna-purification/rna-purification/total-rna/rneasy-plus-kits ) states that “the RNeasy Plus Micro Kit purifies total RNA from up to 5 x 10 ^5^ cells or 5 mg tissue, and the RNeasy Plus Mini Kit isolates total RNA from up to 10 ^7^ cells or 30 mg tissue.” Given this, we chose to use the mini rather than micro kit, and found that RNA isolation rates were consistent provided the tissue disruption and homogenisation steps were followed (described on page 17, “Results and Analytical Methods” subsection “RNA Extraction for RT-qPCR”, figure 6a): “SSBM constructs are disrupted and homogenised prior to extraction of RNA for qPCR analysis, by following a process optimised for these constructs. Disruption involves vortexing samples, without the anchors, with glass beads, then by repeated aspiration using a syringe and needles. Disrupted samples are homogenised using QIAShredders (cat no.: 79656; supplier: QIAGEN), with the resultant sample ready for RNA extraction.”

  

- *I am not convinced that Fig. 7 suggests the presence of osteocytes in the tissue through a single podoplanin-positive staining in the absence of controls as this protein is also expressed on other cell types. IF staining should include other accepted bone-specific or bone maturation makers. This could be easily checked and should be considered. *

**Author Response: **We recognise the reason for the reviewer’s comment. The images in figure 7 were included to evidence that immunostaining of the constructs is possible rather than to draw conclusions about cell differentiation.

 In this manuscript we make the case that this model is capable of long-term culture of osteoblasts and their differentiation towards osteocytes. In response to the reviewer’s comment (and to a similar comment from reviewer 1), we have amended the language within the manuscript to clarify that this method supports the long-term culture of osteoblasts and their differentiation towards osteocytes, but we stop short of stating that mature osteocyte formation occurs.

 Of note, images of mature osteocytes within SSBM constructs have been previously published in Iordachescu et al. 2018, which is referenced frequently throughout the manuscript. Given that the aim of the current manuscript was to provide a detailed and practical methodology for a previously published method (albeit with a different cell source), we consider that these previously published images are sufficient.

  

- *Importantly, while using XRF is a nice idea, demonstrating calcium and phosphorous within the SSBM constructs does not make this a “bone model”. To this end, a major limitation of the present methodology is that no biomineralization has been shown, yet, while the title suggests a “bone model” was developed. Stating that “mineral distribution and maturation appears heterogeneous as found in in vivo bone tissue” does not really convince me. Please provide further evidence.*

**Author Response: **We recognise the reviewer’s concern that a single analytical method is not sufficient to ascertain whether the mineralisation that occurs here is true biomineralisation. However, the intention of this paper is to provide a methodology for creating SSBMs as well as indicating the analytical methods by which they can be characterised biologically and structurally. Additional characterisation using Ramen spectroscopy has previously been published (Iordachescu et al. 2018), strengthening the evidence that biomineralization occurs in the SSBM.

# Reviewer report: Reviewer response for version 1

**DOI:** 10.5256/f1000research.143559.r189266

**Published:** 2024-02-19

**Authors:** Jesus Delgado-Calle, Hayley Sabol

**Affiliations:** 1 University of Arkansas for Medical Sciences, Little Rock, USA

In this manuscript, Finlay and colleagues provide a detailed, step-by-step protocol to establish 3D cultures of osteoblastic cells. The rationale for developing a 3D in vitro system for long culture of osteoblasts and their differentiation to osteocytes is strong. The description of the procedures is thorough and comprehensive. Specific comments to the authors are shown below:

1. The authors indicate this is an inexpensive method doable for most labs. However, the first steps of the process require the use of a 3D printer. The authors need to tone down their statements about how easy and inexpensive this method is. Is there an alternative to the 3D printed mould?
2. HOB-01-C1 is not an osteocytic cell line, is a pre-osteocyte-like cell line.
3. The manuscript would benefit from the inclusion of a deeper discussion of the different 3D models and ex vivo models currently available to study osteocyte biology and a description of the major advantage(s) of using the described system. 
4. The authors suggest this model can substitute in vivo approaches. This statement needs to be toned down, as the system as presented does not represent the complexity of the bone microenvironment. Even a co-culture between osteoblasts and osteoclasts/tumor cells will only represent a minor fraction of the cells involved in bone biology.
5. The reader would benefit from the inclusion of clear visual examples of the morphology of osteocytes generated in this in vitro system.
6. The osteogenic step is not clearly defined. Reagents and concentrations, as well as treatment time, need to be indicated.
7. The protocol seems to take a long time, increasing user error and the chance of contamination. Advice on how to avoid contamination needs to be provided.

Is the rationale for developing the new method (or application) clearly explained?

Yes

Are the 3Rs implications of the work described accurately?

Partly

Is the description of the method technically sound?

Yes

Are the conclusions about the method and its performance adequately supported by the findings presented in the article?

Partly

If any results are presented, are all the source data underlying the results available to ensure full reproducibility?

Yes

Are a suitable application and appropriate end-users identified?

Yes

Are sufficient details provided to allow replication of the method development and its use by others?

Yes

Reviewer Expertise:

osteocyte, bone biology, cancer.

We confirm that we have read this submission and believe that we have an appropriate level of expertise to confirm that it is of an acceptable scientific standard, however we have significant reservations, as outlined above.

# Response

**Published:** 2024-04-29

**Authors:** Amy Naylor

**Affiliations:** University of Birmingham, UK, UK

- *The authors indicate this is an inexpensive method doable for most labs. However, the first steps of the process require the use of a 3D printer. The authors need to tone down their statements about how easy and inexpensive this method is. Is there an alternative to the 3D printed mould? *

**Author Response: **Whilst 3D printing is not available to everyone, it does not require expensive equipment or maintenance in comparison to typical lab equipment and reagents and once trained, is easy to use. Alternatively, there are multiple companies who will print the frame at very low cost e.g., £3 per frame as of March 2024. Furthermore, once the frame is printed, it can be reused multiple times to create many anchor moulds.

 To support the reader in navigating this, we have added the following text to the relevant methods section:

 “Note - if unable to 3D print on site, there are several professional online printing services who can print the frame quickly and cost effectively from the 3D object file. Recommendation to print 1-3 frames."

 "Note - once printed, positive frames can be reused multiple times (until anchor shape has become distorted). Shelf life: >4 years."

  

- *HOB-01-C1 is not an osteocytic cell line, is a pre-osteocyte-like cell line. *

**Author Response: **Agreed, we have changed the wording of the sentence to more correctly reflect this.

  

- *The manuscript would benefit from the inclusion of a deeper discussion of the different 3D models and ex vivo models currently available to study osteocyte biology and a description of the major advantage(s) of using the described system. *

**Author Response: **We agree and have added a section to introduction that expands upon this point and have referenced recent reviews that discuss this in depth.

  

- *The authors suggest this model can substitute in vivo approaches. This statement needs to be toned down, as the system as presented does not represent the complexity of the bone* *microenvironment. Even a co-culture between osteoblasts and osteoclasts/tumor cells will only represent a minor fraction of the cells involved in bone biology.*

**Author Response: **We agree with the reviewer, however it is not our ambition with this model to recapitulate the complexity of the bone microenvironment, rather to create a long-term *in vitro* environment that can successfully support osteoblasts. We have acknowledged in the conclusion that this culture cannot replicate the bone microenvironment in its entirety but that it adds to the *in vitro* options available to researchers in their endeavours to reduce reliance on *in vivo* models, particularly when it comes to the toxicity and pharmacokinetic testing phases. We have also highlighted the properties that can allow our model to be made into more complex *in vitro* models and we encourage the reader to use this method to do further research into this. We have also highlighted the unique feature of our model in the introduction, namely the in-built strain achieved via the anchors, which we believe is crucial to its recapitulating the bone matrix microenvironment.

  

- *The reader would benefit from the inclusion of clear visual examples of the morphology of osteocytes generated in this in vitro system. *

**Author Response: **We recognise the reason for the reviewer’s comment. In this manuscript we make the case that this model is capable of long-term culture of osteoblasts and their differentiation towards osteocytes. In response to the reviewer’s comment, we have amended the language within the manuscript to clarify that this method supports the differentiation of osteoblasts towards osteocytes, but we stop short of demonstrating evidence of mature osteocyte formation. Of note, images of osteocytes within the constructs have been previously published in Iordachescu et al. 2018, albeit with a different cell source, which is referenced frequently throughout the manuscript.

 Given that the aim of this manuscript was to provide a detailed and practical methodology for a previously published method, we consider that these previously published images are sufficient. Of note, we are currently preparing another manuscript that will characterise the cellular biology of hFOB1.19 in SSBM over time in more detail.

  

- *The osteogenic step is not clearly defined. Reagents and concentrations, as well as treatment time, need to be indicated. *

**Author Response: **We agree. Thank you for recommending this clarification. We have edited the table with suggested cell line seeding densities to include suggested osteogenic media, whilst making clear that this is at the discretion of the user, and we have added additional information in section 5 of the methodology.

  

- *The protocol seems to take a long time, increasing user error and the chance of contamination. Advice on how to avoid contamination needs to be provided. *

**Author Response: **Unfortunately, this protocol does indeed take time. However, in our experience there is no greater risk of cell culture contamination than with the maintenance of e.g., cell lines, provided stringent aseptic technique and conditions are adhered to. We have included a list of simple suggestions to reinforce this message, such as frequent decontamination of incubator and culture hood, separate pipettes, regular media filtration etc.
